# Supplementary material for: Prospective randomised trial examining the impact of an educational intervention versus usual care on anticoagulation therapy control based on an SAMe-TT2R2 score-guided strategy in anticoagulant-naïve Thai patients with atrial fibrillation (TREATS-AF): a study protocol
Source: BMJ Open. 2021 Oct 7;11(10):e051987. doi: 10.1136/bmjopen-2021-051987 (PMC8506852; doi:10.1136/bmjopen-2021-051987)
Supplement: Supplementary data [file bmjopen-2021-051987supp001.pdf]

Appendix (Investigators list)

Trial Management Group of TREATS-AF: Arintaya Phrommintikul, Gregory Lip, G Neil Thomas, Rungroj Krittiyaphong, Wanwarang Wongcharoen, Surakit Nathisuwan, Gemma Slinn, Sukhi Sehmi, Siriluck Gunaparn, Deirdre Lane, Kate Jolly, Susan Jowett, Unchalee Permsuwan, Jonathan Mathers, Wichuda Jiraporncharoen, Chaisiri Angkurawaranon, Anita Slade, Samir Mehta, Antika Wongthanee, Neil Winkle

Trial Steering Committee – TSC: Smonporn Boonyaratavej Songmuang – Chair, Rapeephon Kunjara Na Ayudhya, Lin Yenn-Jiang, Tomorn Thongsri

Data Monitoring and Ethics Committee – DMEC: Tippawan Liabsuetrakul, Kemmawadee Preedalikit, Thoranis Chantrarat,

Participating local investigator list of TREATS-AF;

1. Chiangrai Prachanukroh Hospital: Wattana Wongtheptien, Suparat Wattanasombat, Jutarat Kaewdam, Nongluk Mananusorn, Wuttichai Sawatna

2. Nakornping Hospital: Thanyaluck Chotayaporn, Kultida Lertthanaphol, Busarakam Pothongsunun, Jirarat Chandee, Tananya Prasit, Chotika Ruttanapornnukul, Ketsanee Sripattanatrakul, Vullida Cheingyothakul

3. Maharaj Nakorn Chiang Mai Hospital: Narawudt Prasertwitayakij, Kanokporn Niwatananun, Voratima Silavanich, Mantiwee Nimworapan, Phornwinee Somsap, Tikumporn Pornwisrtsirikul, Praphaphan Daoram, Chayanit Srisongmuang

4. Lampang Hospital: Natrawee Bureekam, Siriporn Intharangsri, Chonlada Kawila

5. Siriraj Hospital: Arjbordin Winigkul, Yodying Kaolawanich, Ajjma Sarapakdi, Usanee Pienpattapong, Olaree Chaiphet

6. Maharat Nakhon Ratchasima Hospital: Bancha Sookananchai, Piti Niyomsiriwanich, Weerapan Wiwatworapan, Ubonwan Sapoo, Suttiwanik Kowitphattana, Ketmanee Sae-Ueng, Arisorn Jirapermpun

7. Srinagarind Hospital and Queen Sirikit Heart Center: Vichai Senthong, Supaporn Onsanit, Siriporn Jantharuechai, Wachiraya Tipboonchu
