# Supplementary material for: Prospective randomised trial examining the impact of an educational intervention versus usual care on anticoagulation therapy control based on an SAMe-TT2R2 score-guided strategy in anticoagulant-naïve Thai patients with atrial fibrillation (TREATS-AF): a study protocol
Source: BMJ Open. 2021 Oct 7;11(10):e051987. doi: 10.1136/bmjopen-2021-051987 (PMC8506852; doi:10.1136/bmjopen-2021-051987)
Supplement: Author's manuscript [file bmjopen-2021-051987.draft_revisions.pdf]

BMJ Open

BMJ Open is committed to open peer review. As part of this commitment we make the peer review history of every article we publish publicly available.

When an article is published we post the peer reviewers' comments and the authors' responses online. We also post the versions of the paper that were used during peer review. These are the versions that the peer review comments apply to.

The versions of the paper that follow are the versions that were submitted during the peer review process. They are not the versions of record or the final published versions. They should not be cited or distributed as the published version of this manuscript.

BMJ Open is an open access journal and the full, final, typeset and author-corrected version of record of the manuscript is available on our site with no access controls, subscription charges or pay-per-view fees (http://bmjopen.bmj.com).

If you have any questions on BMJ Open's open peer review process please email info.bmjopen@bmj.com

BMJ Open

Prospective randomised trial examining the impact of an educational intervention versus usual care on anticoagulation therapy control based on a SAMe-TT2R2 score guided strategy in anticoagulant-naïve Thai patients with atrial fibrillation (TREATS-AF): study protocol

Journal:	*BMJ Open*
Manuscript ID	bmjopen-2021-051987
Article Type:	Protocol
Date Submitted by the Author:	09-Apr-2021

Complete List of Authors:	Phrommintikul, Arintaya; Chiang Mai University Faculty of Medicine, Division of Cardiology, Department of Internal Medicine<br>Nathisuwan, S; Mahidol University Faculty of Pharmacy, Department of Pharmacy<br>Gunaparn, Siriluck; Chiang Mai University Faculty of Medicine, Division of Cardiology, Department of Internal Medicine<br>Krittayaphong, Rungroj; Mahidol University Faculty of Medicine Siriraj Hospital<br>Wongcharoen, Wanwarang; Chiang Mai University Faculty of Medicine, Division of Cardiology, Department of Internal Medicine<br>Sehmi, Sukhi; University of Birmingham Clinical Trials Unit<br>Mehta, Samir; University of Birmingham Clinical Trials Unit<br>Winkles, Neil; University of Birmingham Clinical Trials Unit<br>Brocklehurst, Peter; University of Birmingham Clinical Trials Unit<br>Mathers, Jonathan; University of Birmingham Institute of Applied Health Research<br>Jowett, Sue; University of Birmingham Institute of Applied Health Research, Health Economics Unit<br>Jolly, Kate; University of Birmingham Institute of Applied Health Research<br>Lane, Deirdre; University of Liverpool Faculty of Health and Life Sciences, Cardiovascular Health<br>Thomas, Neil; University of Birmingham Institute of Applied Health Research, Department of Public Health and Epidemiology<br>Lip, Gregory; University of Liverpool, Liverpool Centre for Cardiovascular Science

Keywords:	CARDIOLOGY, QUALITATIVE RESEARCH, HEALTH ECONOMICS

SCHOLARONE™
Manuscripts

I, the Submitting Author has the right to grant and does grant on behalf of all authors of the Work (as defined in the below author licence), an exclusive licence and/or a non-exclusive licence for contributions from authors who are: i) UK Crown employees; ii) where BMJ has agreed a CC-BY licence shall apply, and/or iii) in accordance with the terms applicable for US Federal Government officers or employees acting as part of their official duties; on a worldwide, perpetual, irrevocable, royalty-free basis to BMJ Publishing Group Ltd ("BMJ") its licensees and where the relevant Journal is co-owned by BMJ to the co-owners of the Journal, to publish the Work in this journal and any other BMJ products and to exploit all rights, as set out in our licence.

The Submitting Author accepts and understands that any supply made under these terms is made by BMJ to the Submitting Author unless you are acting as an employee on behalf of your employer or a postgraduate student of an affiliated institution which is paying any applicable article publishing charge ("APC") for Open Access articles. Where the Submitting Author wishes to make the Work available on an Open Access basis (and intends to pay the relevant APC), the terms of reuse of such Open Access shall be governed by a Creative Commons licence – details of these licences and which Creative Commons licence will apply to this Work are set out in our licence referred to above.

Other than as permitted in any relevant BMJ Author's Self Archiving Policies, I confirm this Work has not been accepted for publication elsewhere, is not being considered for publication elsewhere and does not duplicate material already published. I confirm all authors consent to publication of this Work and authorise the granting of this licence.

For peer review only

Prospective randomised trial examining the impact of an educational intervention versus usual care on anticoagulation therapy control based on a SAMe-TT_2R_2 score guided strategy in anticoagulant-naïve Thai patients with atrial fibrillation (TREATS-AF): study protocol

Arintaya Phrommintikul*, Surakit Nathisuwan, Siriluck Gunaparn, Rungroj Krittayaphong, Wanwarang Wongcharoen, Sukhi Sehmi, Samir Mehta, Neil Winkles, Peter Brocklehurst, Jonathan Mathers, Sue Jowett, Kate Jolly, Deirdre A. Lane, G Neil Thomas*, Gregory Y. H. Lip*; on behalf of the TREATS-AF Study Group**

[*joint senior authors]

**TREATS-AF Study Group

Narawudt Prasertwitayakij, Wattana Wongtheptien, Thanyaluck Chotayaporn, Natrawee Bureekam, Bancha Sookananchai, Vichai Senthong, Antika Wongthanee, Gemma Slinn, A Slade

Corresponding authors:

G Neil Thomas: gneilthomas@gmail.com/ g.n.thomas@bham.ac.uk

Gregory Lip: Gregory.Lip@liverpool.ac.uk

Running head: Educational intervention for anticoagulation management

Word count: Abstract: 289 words; Main Text: 3,693 words

Tables: 2 Figures: 2

For peer review only

Abstract

Introduction: The burden of atrial fibrillation (AF) in Thailand is high and associated with increased morbidity, mortality and healthcare costs. Vitamin-K antagonists (VKA, e.g. warfarin, commonly used for stroke prevention in AF patients in Thailand, are effective but are often sub-optimally controlled. We aim to evaluate the impact of a SAMe-TT$_2$R$_2$ score guided strategy and educational intervention compared to usual care on anticoagulation control expressed by the time in therapeutic range (TTR) at 12-months, in anticoagulant-naïve Thai patients with AF.

Methods and analysis: Multi-centre, open-label, parallel-group, randomised controlled trial conducted in Thailand among adult patients (age ≥ 18 years) with AF who are anticoagulant-naïve. Patients will be randomised to one of two groups; a SAMe-TT$_2$R$_2$ score guided strategy with educational intervention and usual care vs. usual care alone. The planned follow-up period is 12 months. The primary outcome is time in therapeutic range (TTR) at 12 months.Secondary outcomes include: 1) TTR at 6 months; 2) thromboembolic and bleeding events at 12 months; 3) composite major adverse cardiovascular events (MACE) at 12 months; 4) change in patients' knowledge of AF between baseline and 6 months and 12 months; 5) cost-effectiveness; 6) quality-of-life at baseline, 6 and 12 months using EQ-5D-5L (Thai version); and 7) patient satisfaction/perceptions of the TREAT intervention. An embedded qualitative study will assess patient perceptions of the TREAT intervention.

Ethics and dissemination: The study has been approved by the Ethical Review Committee, Ministry of Public Health of Thailand and registered in The Thai Clinical Trials Registry (TCTR20180711003). The results of this trial will be submitted for publication in a peer-reviewed journal. Participants will be informed via a link to a preview of the publication. A lay summary will also be provided to all participants prior to publication.

Keywords: anticoagulants, atrial fibrillation, SAMe-TT2R2 score, warfarin

For peer review only

Strengths and limitations of this study

- Warfarin is the default therapy for stroke prevention in atrial fibrillation (AF) in several countries, but is often sub-optimally managed.
- A one-time structured educational-behavioural intervention (TREAT intervention) among those predicted to be less likely to achieve optimal control, may be a simple and cost-effective adjunct management strategy which could help improve time in therapeutic range**.**
- This prospective individually-randomised-controlled trial (RCT) in anticoagulant-naïve patients with AF is conducted to assess whether the application of a SAMe-TT_2R_2 score-guided strategy and TREAT intervention plus usual care could improve anticoagulation control with warfarin.
- The study design is comprehensive with multidisciplinary team approach. The efficacy, safety, patients' satisfaction as well as cost-effectiveness will be assessed.

For peer review only

Introduction

Atrial fibrillation (AF) is associated with decreased quality of life, and increased mortality and morbidity from stroke/thromboembolism.[1 2] Incidence of AF has been increasing in developing countries including Thailand, partly due to the rapid change of population dynamics towards an ageing society.[3 4] The hospitalisation rate for Thai AF patients was high (15.5 per 100,000 person-years), with a high mortality (44.0% after 72-month follow-up (average 46 months)).[5] The high burden of AF in Thailand is also associated with similarly high healthcare costs[6].

Appropriate oral anticoagulation, either with a vitamin-K antagonist (VKA, e.g. warfarin) or non-VKA oral anticoagulants (NOAC), is essential for effective stroke prevention in AF patients with one or more stroke risk factors.[7] Despite having various advantages over VKA, prohibitive costs means access to NOACs are generally limited for low-to-middle income countries such that VKAs are widely used globally[8 9]. In Thailand, warfarin is the default therapy for stroke prevention in AF, but is often sub-optimally managed due to the many inherent limitations associated with this drug, including diet-, drug-, and alcohol-interactions and difficulties with anticoagulation monitoring. Recent data from retrospective cohort studies, along with a nationwide registry, suggested that the average TTR among AF patients in Thailand ranged from 50 to 55%. [10-13] Those with sub-optimal anticoagulation control, TTR<65%, had a 2 to 3-fold increased risk of stroke, major bleeding, and death compared to those with TTR ≥65%.[12] As a result, strategies to improve TTR control are clearly needed and would likely have a major impact on preventing these adverse events.

TTR is influenced by numerous modifiable and non-modifiable factors, but the more common clinical factors have been used to formulate the SAMe-TT$_2$R$_2$ score (Table 1).[14]

Table 1. Components of the SAMe-TT$_2$R$_2$ score

Components		Points
**S**	Sex (female)	1
**A**	Age (<60 years)	1
**Me**	Medical history*	1
**T**	Treatment (interacting drug e.g., amiodarone)	1

For peer review only - http://bmjopen.bmj.com/site/about/guidelines.xhtml

**T**	Tobacco use (within 2 years)	2
**R**	Race (non-Caucasian)	2
Maximum total		8
* More than two of the following: hypertension, diabetes mellitus, coronary artery disease/myocardial infarction, peripheral atrial disease, congestive heart failure, previous stroke, pulmonary disease, hepatic or renal disease		

SAMe-TT2R2 scores 0-2; the patients with AF likely to achieve and maintain optimal TTR and SAMe-TT2R2 > 2; the patients with AF likely poorer responders.

Several observational studies including those conducted in Asian populations have demonstrated that a SAMe-TT_2R_2 value of >2 is predictive of poor TTR, all-cause mortality and the composite end-point of thromboembolic events, major bleeding and mortality.[13 15-17] By identifying those who are at risk of poor TTR, targeted efforts to improve TTR can be effectively provided to the vulnerable group. If TTR can be improved to >65%, VKAs can provide comparable efficacy and safety to that seen with NOACs.[18 19] Previous studies have demonstrated that many patients with AF possess little knowledge about AF and do not understand the risks/benefits of OAC,[20-25] and this may contribute to poor INR control.

We have previously shown that an educational-behavioural intervention (TREAT, ISRCTN93952605) significantly improved TTR 6-months after warfarin initiation compared to usual care alone (78.5% vs. 66.7% respectively; p=0.01).[26-28] Thus, a one-time structured educational-behavioural intervention among those predicted to be less likely to achieve good INR control, may be a simple and cost-effective adjunct management strategy which could help improve individual TTR. Increased patient understanding of disease and treatment, and a reduction of adverse events, may improve quality of life.

We therefore conducted a prospective individually-randomised-controlled trial (RCT) in anticoagulant-naïve Thai patients with AF to assess whether the application of a SAMe-TT_2R_2 score-guided strategy and TREAT intervention[26-28] plus usual care could improve anticoagulation control

For peer review only

with warfarin (measured by percentage TTR) at 12 months (primary outcome) compared with usual care alone.

Objectives

The primary objective of this study is to evaluate the impact of a SAMe-TT_2R_2 score guided strategy and educational intervention (plus usual care) compared to usual care alone, on patient's anticoagulation control with warfarin, as measured by the time in therapeutic range (TTR) at 12 months, in anticoagulant-naïve Thai patients with AF. As secondary objectives, we aim to evaluate the impact of this intervention on patient knowledge, TTR at 6 months, thromboembolic and bleeding events and the composite major adverse cardiovascular events (MACE). We will also perform analysis to evaluate the cost-effectiveness of the intervention and investigate patients' satisfaction and acceptance of the TREAT intervention using a brief questionnaire and further in a qualitative sub-study.

Methods

Study design & setting

This is a multi-centre, open-label, paralleled-group, RCT conducted among adult Thai patients (age ≥18 years) with AF who are anticoagulant-naïve. Patients will be randomised to one of two groups, either a SAMe-TT_2R_2 score guided strategy (see Table 1) and educational intervention plus usual care vs. usual care alone. The planned follow-up period is 12 months. Study sites are seven public, hospitals across six provinces (Bangkok, Chiang Mai, Chiang Rai, Lampang, Khon Kaen and Nakhon Ratchasima) in Thailand. To promote generalisability of our findings, these seven hospitals are a combination of three university hospitals (Maharaj Nakorn Chiang Mai Hospital, Siriraj Hospital, and Queen Sirikit Heart Center of the Northeast) and four secondary/tertiary care hospitals (Maharat Nakhon Ratchasima Hospital, Chiang Rai Prachanukroh Hospital, Nakornping Hospital, and Lampang Hospital). The study protocol was approved by the Institutional Ethical Committee of each study site and by the IRB of the Ministry of Public Health, the Royal Government of Thailand

For peer review only

(Central Research Committee (CREC) No. COA-CREC 007/2020) and registered with the Thai Clinical Trials Registry reference number: TCTR20180711003).

Participants

Newly diagnosed adult AF patients identified and referred from cardiology, internal medicine, family medicine and general clinics at the hospitals and referrals from primary care will be eligible for enrolment into the study.

Eligibility Criteria

Patient must meet all of the following inclusion criteria to be eligible for randomisation:

(1) ≥18 years of age

(2) Newly diagnosed non-valvular AF patients

(3) ECG-documented AF

(4) Warfarin-eligible (men with CHA_2DS_2VASc score ≥1; women with CHA_2DS_2VASc score ≥2)

(5) Warfarin-naïve (no treatment with anticoagulation within the past 12 months, treatment may have started within the prior 28 days from randomisation

(6) Able to comply with scheduled visits, treatment plan and laboratory tests

(7) Able to give informed consent and comply with study protocol (with support of a carer)

Patients will be excluded from participation if they present with any of the following:

(1) Any contraindication to oral anticoagulation

(2) Prosthetic cardiac valve or significant valvular heart disease with an indication for heart surgery

(3) Likelihood of intermittent or permanent discontinuation of warfarin during follow-up (e.g., Major surgery or post-AF ablation)

(4) Known active malignancy with a life expectancy less than 5 years

(5) Diagnosed significant cognitive impairment preventing provision of informed consent and/or able to comply with the study protocol

For peer review only

(6) Any disease likely to cause death within 12 months

Randomisation

After participant eligibility has been confirmed and informed consent has been provided, the participant will be randomised into the trial. Randomisation will be done using a web-based platform with blinded allocation. Randomisation will be stratified based on centre, sex (male or female) and baseline SAMe-TT_2R_2 score (0-2, 3-5, and 6-8). Participants will be randomised at the level of the individual in a 1:1 ratio to either the SAMe-TT_2R_2 score guided strategy and educational intervention plus usual care (intervention group) or usual care alone. (Figure 1 and 2) Group 1 is the usual care group using warfarin (control group). For Group 2, the patients are further divided into two groups. Group 2a are patients with a SAMe-TT_2R_2 score 0-2 who will receive warfarin plus usual care. Group 2b are patients with SAMe-TT_2R_2 score >2 group who will receive warfarin plus the TREAT education-behavioural intervention as an adjunct to their regular INR monitoring, to improve their TTR.

Trial intervention

As described previously, the TREAT intervention is a patient-centred intervention for patients with AF co-developed through the integration of theoretical and clinical frameworks, and patient feedback.[26 27] This behaviour-change intervention package consists of an educational booklet, diary, worksheet and a DVD for reinforcement. The educational booklet covers AF causes and consequences, warfarin and its metabolism, stroke risk and risk of bleeding on treatment and lifestyle changes (diet, alcohol, lifestyle change). The patient DVD contains health professionals and 'expert patient' narratives that covers AF causes, consequences, side effects, treatment options, warfarin, INR monitoring, lifestyle changes, along with common psychological and physical barriers to anticoagulation that patients may experience. The patient worksheets include a simple self-calculation of stroke risk (CHA_2DS_2-VASc score), personal barriers to uptake of warfarin, and discussion of personal goals for lifestyle changes. In addition, a self-monitoring diary including diet, alcohol intake (in units), warfarin regimen and INR results is also provided.

To overcome the language barrier of the general Thai public toward English, the TREAT intervention was culturally adapted to fit the local context and then translated. Forward and backward translation

For peer review only

by two bilingual experts was performed. A test run of the Thai-version of the TREAT intervention was conducted among AF patients with a range of educational ability (Thai literacy level is 94%) and age levels. Patient feedback was incorporated to improve the usability of the Thai-version TREAT intervention.

A training session was conducted to prepare all study site coordinators (pharmacists and nurses) on how to deliver the intervention effectively. To avoid contamination, those who provide the TREAT intervention and those who provide usual care will be two different groups of pharmacists and nurses. The intervention will be delivered within 4-weeks of initiating warfarin. Intervention fidelity will be ensured by direct observation of random sessions at each centre by the trainer. Patients in the usual care group will receive training on the OAC management from the anticoagulation clinic, and patient information about AF and need for warfarin by a healthcare professional using the standard warfarin education checklist.

Patient and Public Involvement

Patients or the public were not involved in the design, or conduct, or reporting, or dissemination plans of our research. The patients involved in the development of Thai-version of the TREAT intervention.

Outcome measures

The primary outcome is time in therapeutic range (TTR) at 12 months. Secondary outcomes include 1) TTR at 6-months; 2) thromboembolic and bleeding events from at 12 months; 3) composite major adverse cardiovascular events (MACE) outcome of non-fatal MI, non-fatal stroke and cardiovascular death and all-cause mortality, in an exploratory analysis (combined and individually) at 12 months; 4) change in patients' knowledge of AF from baseline to 6 months and 12 months; 5) cost-effectiveness; 6) quality-of-life at baseline, 6 and 12 months assessed using EQ-5D-5L (Thai version)[29 30]; and 7) patient satisfaction/perceptions of the TREAT intervention. An embedded qualitative study will assess patient perceptions of the TREAT intervention.

Study procedures and schedules of assessment

Baseline SAMe-TT$_2$R$_2$ score will be calculated (Table 1). All patients will receive warfarin, dose-adjusted to achieve a target INR of 2.0 to 3.0. INR monitoring will be undertaken at routine intervals

For peer review only

(as would occur in usual care) and will be recorded over a 12-month period. Patients will be randomised as described in Figure 1, either to Group 1 (usual care) or Group 2 (intervention arm), for which the trial is powered. Group 2 is a directed management group based on a: patients with AF likelihood of achieving and maintaining optimal TTR (SAMe-TT_2R_2 scores 0-2; Group 2a) and those likely to achieve poor TTR (SAMe-TT_2R_2 scores >2, Group 2b). From our prior data, approximately 15%-20% will likely enter Group 2a. The primary and secondary outcomes between Group 1 and Group 2 (together) will be compared. The schedule of assessment is presented in Table 2. INR monitoring is independent of the intervention (i.e. naturalistic) as we recognise that the improvement of TTR may be a result of more frequent monitoring. Patients in all groups will have their INR monitored as they would if they were not in the trial (by anticoagulation services which are separate from the trial and who are blinded to treatment allocation).

Table 2. Study visits schedule

CLINICAL ASSESSMENT:

A. Demographic information: age, sex, ethnicity, education level, height (cm), weight (kg), body mass index, smoking status, alcohol use.
B. Medical history: history of arterial hypertension, heart failure, diabetes mellitus, coronary artery disease (myocardial infarction, PCI, CABG), peripheral artery disease, stroke, transient ischemic attack, systemic embolism, COPD, chronic kidney disease, chronic liver disease, thyroid disease, bleeding event
C. Physical exam, blood pressure, heart rate, EHRA and NYHA class
D. Concomitant medical therapy

OAC THERAPY EDUCATION: ALL PATIENT-a standard OAC education session conducted by a healthcare professional about the clinical significance and management of OAC therapy

QUESTIONNAIRES: Atrial fibrillation knowledge questionnaires, patient satisfaction (Likert scale) and EQ-5D-5L (Thai version)

HEALTH ECONOMICS ASESSMENT: EQ-5D-5L (Thai version), AF, warfarin and clinical event-related healthcare resource use, intervention costs

FOLLOW-UP OF CLINICAL EVENTS: TTR; composite of thromboembolism, major bleeding, all-cause death (in all patients).

	**BASELINE**	**M1±7D**	**M2±7D**	**M6±14D**	**M12±14D**
**CLINICAL ASSESSMENT**	**a,b,c,d**	**c**	**c**	**c**	**c,d**
**ECG**	x				

For peer review only

**INR†**	x	x	x	x	x
**STANDARD OAC THERAPYEDUCATION**	x				
**TREAT intervention**		x			
**QUESTIONNAIRES**	x			x	x
**QUALITATIVE ASSESSMENT**	x‡			x	
**HEALTH ECONOMIC ASSESSMENT**	x			x	x
**FOLLOW-UP OF CLINICAL EVENTS**				x	x
**†INR MONITTORING IS MANDATORY** during the 12 month follow-up period for all patients, with frequency as per usual care conducted by anticoagulation clinics<br>‡Baseline interviews of patients will take place within 4 weeks of receiving the intervention					

Data collection and management

Medical records of the participating hospitals serve as the source data including clinical information and laboratory values which are accessible and maintained in accordance to national standards and regulations. Outcome data will be extracted from participant's clinical notes and laboratory reports into an electronic CRF. For long term storage, clinical data will be electronically archived at Faculty of Medicine, Chiang Mai University, Thailand as per the regulatory requirements for Good Clinical Practice and in line with the Medical Research Council (MRC) requirements and local policies.

The questionnaires will be completed by the patient. For those with literacy problems, the questionnaires will be read out to the participant, the participant will respond to each question and their responses will be recorded by the staff member administering the questionnaires.

Trial data will be captured in a REDCAP, secure SQL server database. An independent Data Monitoring Committee (DMC), including both local and international experts, has been appointed and convened. Interim analyses of major outcome measures and safety data will be conducted and provided in strict confidence to the DMC. Any decision to stop the trial early will be based on the balance of efficacy and safety.

Sample size and power calculations

For peer review only

Most recent studies suggest that the mean TTR in Thai patients receiving warfarin is in the range of 50%-55%.[11 12] Since an improvement of 10% in the mean TTR is likely to be clinically meaningful, we assume a 10% difference in 12-month TTR with 90% power and 5% significance level for our sample size calculation and using a standard deviation of “26” as observed in a previous Thai study to determine the sample size estimates. Hence to detect a 10% mean difference in TTR at 12 months, using two-sided test, sample size for randomisation in a 1:1 ratio would be 288 or 144 per arm. Assuming and adjusting for a 10% attrition/loss to follow-up rate, a total sample size of 320 patients or 160 per arm is needed.

Statistical analysis

Continuous variables will be presented as mean (SD), or median (interquartile range, IQR), as appropriate; categorical variables will be reported as counts with percentages. Descriptive statistics will be presented for baseline demographic and clinical information. For all major outcomes, summary statistics and differences between groups (e.g. mean differences, relative risks) will be presented, with confidence intervals and p-values from two-sided tests also given.

All analyses will be based on the intention to treat (ITT) principle. The primary comparison groups will be composed of those randomised to Group 1, usual care versus those randomised to Group 2, the SAMe-TT_2R_2 guided strategy with the TREAT intervention (plus usual care). All outcomes will be adjusted for the stratification variables (centre, sex and baseline SAMe-TT_2R_2 score) where possible. Two tailed p-values <0.05 will be considered statistically significant. No adjustment for multiple comparisons will be made.

Sub-group analysis

Sub-group analyses will be conducted for the stratification variable sex only. All other stratification variables will be used only to ensure that the two groups are balanced in terms of these important variables. Sub-group analyses will be limited to the primary outcome only. Tests for statistical heterogeneity (e.g. by including the treatment group by subgroup interaction parameter in the regression model) will be performed prior to any examination of effect estimates within subgroups. The results of sub-group analyses will be treated with caution and will be used for the purposes of hypothesis generation only.

For peer review only - http://bmjopen.bmj.com/site/about/guidelines.xhtml

Missing Data and Sensitivity Analyses

Missing and ambiguous data will be queried using a data clarification system, and will focus on data required for trial outcome analysis and safety reporting. Every attempt will be made to collect full follow-up data on all study participants. A sensitivity analysis will be performed on the primary outcome measure only to examine the possible impact of any missing data for the primary outcome. This will include the use of multiple imputation using chained equations. Further sensitivity analysis will also be done if a substantial number of participants do not comply with randomised allocated group and so a per-protocol analysis may also be conducted. Any sensitivity analyses will not, irrespective of their differences, supplant the planned primary analyses.

Interim Analysis

Interim analyses of safety and efficacy for presentation to the independent DMC will take place during the study. The committee will meet prior to study commencement to agree the manner and timing of such analyses. The analysis of the primary and major secondary outcomes and full assessment of safety (adverse events and serious adverse events) will be performed at annual intervals. Criteria for stopping or modifying the study based on this information will be ratified by the DMC.

Planned Final Analyses

The final analysis for the study will occur once the last randomised participant has completed the 12 month follow-up and any corresponding outcome data has been entered onto the study database and validated as being ready for analysis.

Withdrawal

Patients may withdraw consent from the study at any time. Patients may also withdraw from trial treatment but continue with study follow-up and data collection as per the protocol. If the withdrawal is initiated by a health care professional, full details for the reason for withdrawal will be recorded on the case report forms. In all other cases, a simple statement reflecting the patient's preference will be noted.

Analysis of outcome measures

Primary outcome

For peer review only

The primary outcome is the TTR at 12 months and differences in TTR between the intervention and usual care groups will be examined using a linear regression model. Results will be presented as mean difference and 95% confidence interval. A key assumption for this analysis is that the data will be normally distributed; this assumption will be examined visually based on the residuals from the fitted model. If data is deemed not to be normally distributed, then the Mann Whitney U test will be used for analysis.

Secondary outcomes

The secondary endpoints for the trial includes continuous, categorical and time-to-event data items. Any secondary endpoints that are continuous in nature will be analysed in the same way as described for the primary outcome. For dichotomous secondary endpoints, the proportion of participants and percentages will be compared between arms using a log-binomial model. Relative risks and 95% confidence intervals will be calculated. For any time-to-event outcomes these will be compared between treatment arms by using survival analysis methods. Cox proportional hazards model will be used and treatment effects will be expressed as hazard ratios with 95% confidence intervals. Kaplan-Meier survival curves will be constructed for visual presentation of time-to-event comparisons.

Qualitative study

Qualitative data will be obtained directly from patient interviews and interviews with healthcare professionals delivering the intervention as part of an embedded qualitative sub-study. A purposive sample of 10-15 participants from the intervention arm will take part in one-to-one semi-structured interviews with a qualitative researcher, at two time-points (within 4 weeks of the intervention and at 6-months follow-up). Interviews will explore participants' experience of AF; of receiving the TREAT intervention; their perspectives on the utility of different interventions components; as well as behavioural modifications resulting from the intervention. Staff delivering the intervention at each study site will also be interviewed to understand their perceptions of the TREAT intervention and their experience of delivering it to patients. A thematic analysis of interview content will be informed by the

For peer review only

Framework analytic approach. Audio-recorded interviews will be translated and transcribed into English in order to facilitate team-based analysis by Thai and English research team members[31].

Health economics analysis

The economic evaluation will determine the cost-effectiveness of the intensive educational intervention versus usual care. The evaluation will take the form of an incremental cost-utility analysis to estimate cost per quality adjusted life year (QALY) over 12 months follow up from a Thailand health service perspective. If differences in TTR are achieved in the trial, a longer-term Markov model-based cost per QALY analysis will be also undertaken.

Data collection

Data will be collected on health care utilisation required for the intervention, warfarin therapy and monitoring, and health care visits, hospitalisations, investigations, and medication related to thromboembolism, bleeding and management of AF. Information will be collected at the 6 and 12-month patient visits from trial CRFs and hospital records. Additional costings, such as those associated with transportation to the hospital, will be collected from the patients. The EQ-5D-5L (Thai version) questionnaire will be administered to participants at baseline, 6, and 12 months to estimate QALYs.

Data analysis

Unit costs from standard Thai sources will be applied to all health care resource use items, and mean resource use (for each category of health care usage) and mean total costs will be calculated for both trial arms. The current Thailand value set will be applied to patient EQ-5D-5L responses to obtain utility scores and QALYs will be calculated using the area under the curve approach. Multiple imputation will be used to impute all missing values for the EQ-5D-5L and total cost estimates for non-responders. As cost and QALY data is likely to have a skewed distribution, a non-parametric comparison of means (using bootstrapping) will be undertaken. A cost-consequence analysis will initially be reported, describing all the important results relating to resource use, costs and consequences. Incremental cost-effectiveness and cost-utility analyses will then be undertaken to estimate the incremental cost per QALY gained at 12 months, with adjustment for baseline covariates.

For peer review only

The robustness of the results will be explored using sensitivity analysis. Cost-effectiveness acceptability curves (CEACs) will also be produced to reflect the probability the intervention will be cost effective at different cost per QALY willingness to pay thresholds.

Model-based analysis

If the trial data shows a benefit in TTR due to the intervention, trial results will be extrapolated using a Markov model-based cost per QALY. This model will take into account the long-term impact of anticoagulant control on thromboembolism and bleeding over the patient's lifetime, with costs and outcomes discounted at 3%. The model structure will be informed by previously published modelling studies and expert opinion. Extensive deterministic sensitivity analysis using pre-defined ranges will be undertaken to explore which model parameters have most impact on cost-effectiveness. Parameter estimates will be incorporated into the model as distributions to allow for a probabilistic sensitivity analysis, with CEACs constructed to assess the probability of cost effectiveness at different cost per QALY willingness to pay thresholds.

Trial status

The recruitment of participants started in 31st January 2020. Last patient recruitment is expected in May 2021. Data collection is expected to be completed in December 2021.

Dissemination

Results of this trial will be submitted for publication in a peer-reviewed journal. The manuscript will be prepared by the principal investigators or their delegates and authorship will be determined by the trial publication policy. Participants will be informed of the outcome of the trial via a link to a preview of the publication. A lay summary will also be provided via email or posted to participants prior to publication.

For peer review only

Acknowledgments

We acknowledge the assistance and facilities provided by the Medical Research Council (MRC) / Birmingham Clinical Research Facility.

Funding information:

This study is supported by the Newton Fund through the collaboration of the Medical Research Council (MRC), the United Kingdom, the grant reference number is MR/R020892/1 and the Thailand Research Fund (TRF), Thailand, and the grant reference number is DBG6180009.

Authors' contributions:

AP, NT, GL initiated the study. AP is the principal investigator of RCT. SG is the clinical trial manager. AP,NT,SN and GL led the protocol development with contribution from SN, SG, RK, WW, GS, SS, SM, NW, PB, JM, SJ, CJ, DL, KJ. SN, WW and NP prepared educational content including educational booklet, diary, worksheet and a DVD. SN conducted TREAT intervention training for pharmacists and monitored during the trial. AP, WW, RK conduct intervention activities together with research assistants. AW will perform the data analysis. All authors read, contributed to and approved the final version of the manuscript.

Conflict of interest statement:

All other authors have no actual or potential conflict of interest capable of influencing judgment on the part of any author on this work.

For peer review only

AP: reports speaker fees from Bayer, Pfizer, Boehringer Ingelheim, and Daiichi-Sankyo outside the submitted work.

WW: reports speaker fees from Bayer, Pfizer, Boehringer Ingelheim, and Daiichi-Sankyo outside the submitted work.

DL: reports investigator-initiated educational grants from Bristol-Myers Squibb (BMS), speaker fees for Boehringer Ingelheim, Bayer, and BMS/Pfizer and consultancy for BMS/Pfizer, Boehringer Ingelheim, and Daiichi-Sankyo, all outside of the submitted work.

GL: reports consultancy and speaker fees for BMS/Pfizer, Boehringer Ingelheim, and Daiichi-Sankyo. No fees are received personally.

Data sharing: Data are available upon reasonable request.

For peer review only

Reference

1. Hindricks G, Potpara T, Dagres N, et al. 2020 ESC Guidelines for the diagnosis and management of atrial fibrillation developed in collaboration with the European Association of Cardio-Thoracic Surgery (EACTS). *Eur Heart J* 2020 doi: 10.1093/eurheartj/ehaa612 [published Online First: 2020/08/30]

2. Lip GYH, Banerjee A, Boriani G, et al. Antithrombotic Therapy for Atrial Fibrillation: CHEST Guideline and Expert Panel Report. *Chest* 2018;154(5):1121-201. doi: 10.1016/j.chest.2018.07.040 [published Online First: 2018/08/26]

3. Chugh SS, Havmoeller R, Narayanan K, et al. Worldwide epidemiology of atrial fibrillation: a Global Burden of Disease 2010 Study. *Circulation* 2014;129(8):837-47. doi: 10.1161/CIRCULATIONAHA.113.005119 [published Online First: 2013/12/19]

4. Phrommintikul A, Detnuntarat P, Prasertwitayakij N, et al. Prevalence of atrial fibrillation in Thai elderly. *J Geriatr Cardiol* 2016;13(3):270-3. doi: 10.11909/j.issn.1671-5411.2016.03.002 [published Online First: 2016/04/23]

5. Apiyasawat S, Tangcharoen T, Wisaratapong T, et al. CHA(2)DS(2)-VASc scores predict mortality after hospitalization for atrial fibrillation. *Int J Cardiol* 2015;185:293-6. doi: 10.1016/j.ijcard.2015.03.180 [published Online First: 2015/03/31]

6. Ng SS, Nathisuwan S, Phrommintikul A, et al. Cost-effectiveness of warfarin care bundles and novel oral anticoagulants for stroke prevention in patients with atrial fibrillation in Thailand.

For peer review only

Thromb Res 2020;185:63-71. doi: 10.1016/j.thromres.2019.11.012 [published Online First: 2019/11/27]

7. Kirchhof P, Benussi S, Kotecha D, et al. 2016 ESC Guidelines for the management of atrial fibrillation developed in collaboration with EACTS. *Europace : European pacing, arrhythmias, and cardiac electrophysiology : journal of the working groups on cardiac pacing, arrhythmias, and cardiac cellular electrophysiology of the European Society of Cardiology* 2016;18(11):1609-78. doi: 10.1093/europace/euw295 [published Online First: 2016/11/04]

8. Krittayaphong R, Phrommintikul A, Ngamjanyaporn P, et al. Rate of anticoagulant use, and factors associated with not prescribing anticoagulant in older Thai adults with non-valvular atrial fibrillation: A multicenter registry. *J Geriatr Cardiol* 2019;16(3):242-50. doi: 10.11909/j.issn.1671-5411.2019.03.004 [published Online First: 2019/05/14]

9. Tse HF, Wang YJ, Ahmed Ai-Abdullah M, et al. Stroke prevention in atrial fibrillation--an Asian stroke perspective. *Heart Rhythm* 2013;10(7):1082-8. doi: 10.1016/j.hrthm.2013.03.017 [published Online First: 2013/03/19]

10. Saokaew S, Sapoo U, Nathisuwan S, et al. Anticoagulation control of pharmacist-managed collaborative care versus usual care in Thailand. *Int J Clin Pharm* 2012;34(1):105-12. doi: 10.1007/s11096-011-9597-8 [published Online First: 2011/12/29]

11. Priksri W, Rattanavipanon W, Saejear W, et al. Incidence, risk factors, and outcomes of warfarin-associated major bleeding in Thai population. *Pharmacoepidemiol Drug Saf* 2019;28(7):942-50. doi: 10.1002/pds.4781 [published Online First: 2019/05/09]

12. Krittayaphong R, Chantrarat T, Rojjarekampai R, et al. Poor Time in Therapeutic Range Control is Associated with Adverse Clinical Outcomes in Patients with Non-Valvular Atrial Fibrillation: A Report from the Nationwide COOL-AF Registry. *J Clin Med* 2020;9(6) doi: 10.3390/jcm9061698 [published Online First: 2020/06/06]

13. Zulkifly H, Lip GYH, Lane DA. Use of the SAMe-TT2R2 score to predict anticoagulation control in atrial fibrillation and venous thromboembolism patients receiving vitamin K antagonists: A

For peer review only

review. *Heart Rhythm* 2018;15(4):615-23. doi: 10.1016/j.hrthm.2017.11.026 [published Online First: 2017/11/29]

14. Apostolakis S, Sullivan RM, Olshansky B, et al. Factors affecting quality of anticoagulation control among patients with atrial fibrillation on warfarin: the SAMe-TT(2)R(2) score. *Chest* 2013;144(5):1555-63. doi: 10.1378/chest.13-0054 [published Online First: 2013/05/15]

15. Lip GYH, Haguenoer K, Saint-Etienne C, et al. Relationship of the SAMe-TT(2)R(2) score to poor-quality anticoagulation, stroke, clinically relevant bleeding, and mortality in patients with atrial fibrillation. *Chest* 2014;146(3):719-26. doi: 10.1378/chest.13-2976 [published Online First: 2014/04/12]

16. Bernaitis N, Ching CK, Chen L, et al. The Sex, Age, Medical History, Treatment, Tobacco Use, Race Risk (SAMe TT2R2) Score Predicts Warfarin Control in a Singaporean Population. *J Stroke Cerebrovasc Dis* 2017;26(1):64-69. doi: 10.1016/j.jstrokecerebrovasdis.2016.08.030 [published Online First: 2016/09/28]

17. Chan PH, Hai JJ, Chan EW, et al. Use of the SAMe-TT2R2 Score to Predict Good Anticoagulation Control with Warfarin in Chinese Patients with Atrial Fibrillation: Relationship to Ischemic Stroke Incidence. *PLoS One* 2016;11(3):e0150674. doi: 10.1371/journal.pone.0150674 [published Online First: 2016/03/25]

18. Sjogren V, Grzymala-Lubanski B, Renlund H, et al. Safety and efficacy of well managed warfarin. A report from the Swedish quality register Auricula. *Thromb Haemost* 2015;113(6):1370-7. doi: 10.1160/TH14-10-0859 [published Online First: 2015/02/27]

19. Bjorck F, Renlund H, Lip GY, et al. Outcomes in a Warfarin-Treated Population With Atrial Fibrillation. *JAMA Cardiol* 2016;1(2):172-80. doi: 10.1001/jamacardio.2016.0199 [published Online First: 2016/07/22]

20. Lip GY, Kamath S, Jafri M, et al. Ethnic differences in patient perceptions of atrial fibrillation and anticoagulation therapy: the West Birmingham Atrial Fibrillation Project. *Stroke* 2002;33(1):238-42. doi: 10.1161/hs0102.101817 [published Online First: 2002/01/10]

21. Lane DA, Aguinaga L, Blomstrom-Lundqvist C, et al. Cardiac tachyarrhythmias and patient values and preferences for their management: the European Heart Rhythm Association

For peer review only

(EHRA) consensus document endorsed by the Heart Rhythm Society (HRS), Asia Pacific Heart Rhythm Society (APHRS), and Sociedad Latinoamericana de Estimulacion Cardiaca y Electrofisiologia (SOLEACE). *Europace : European pacing, arrhythmias, and cardiac electrophysiology : journal of the working groups on cardiac pacing, arrhythmias, and cardiac cellular electrophysiology of the European Society of Cardiology* 2015;17(12):1747-69. doi: 10.1093/europace/euv233 [published Online First: 2015/06/26]

22. Frankel DS, Parker SE, Rosenfeld LE, et al. HRS/NSA 2014 Survey of Atrial Fibrillation and Stroke: Gaps in Knowledge and Perspective, Opportunities for Improvement. *J Stroke Cerebrovasc Dis* 2015;24(8):1691-700. doi: 10.1016/j.jstrokecerebrovasdis.2015.06.026 [published Online First: 2015/08/11]

23. McCabe PJ, Schad S, Hampton A, et al. Knowledge and self-management behaviors of patients with recently detected atrial fibrillation. *Heart Lung* 2008;37(2):79-90. doi: 10.1016/j.hrtlng.2007.02.006 [published Online First: 2008/03/29]

24. Desteghe L, Engelhard L, Raymaekers Z, et al. Knowledge gaps in patients with atrial fibrillation revealed by a new validated knowledge questionnaire. *Int J Cardiol* 2016;223:906-14. doi: 10.1016/j.ijcard.2016.08.303 [published Online First: 2016/09/03]

25. Lane DA, Ponsford J, Shelley A, et al. Patient knowledge and perceptions of atrial fibrillation and anticoagulant therapy: effects of an educational intervention programme. The West Birmingham Atrial Fibrillation Project. *Int J Cardiol* 2006;110(3):354-8. doi: 10.1016/j.ijcard.2005.07.031 [published Online First: 2005/10/29]

26. Clarkesmith DE, Pattison HM, Lip GY, et al. Educational intervention improves anticoagulation control in atrial fibrillation patients: the TREAT randomised trial. *PLoS One* 2013;8(9):e74037. doi: 10.1371/journal.pone.0074037 [published Online First: 2013/09/17]

27. Clarkesmith DE, Pattison HM, Borg Xuereb C, et al. Developing a Complex Educational-Behavioural Intervention: The TREAT Intervention for Patients with Atrial Fibrillation. *Healthcare (Basel)* 2016;4(1) doi: 10.3390/healthcare4010010 [published Online First: 2016/07/16]

For peer review only

28. Smith DE, Xuereb CB, Pattison HM, et al. TRial of an Educational intervention on patients' knowledge of Atrial fibrillation and anticoagulant therapy, INR control, and outcome of Treatment with warfarin (TREAT). *BMC Cardiovasc Disord* 2010;10:21. doi: 10.1186/1471-2261-10-21 [published Online First: 2010/05/22]

29. Pattanaphesaj J, Thavorncharoensap M, Ramos-Goni JM, et al. The EQ-5D-5L Valuation study in Thailand. *Expert Rev Pharmacoecon Outcomes Res* 2018;18(5):551-58. doi: 10.1080/14737167.2018.1494574 [published Online First: 2018/06/30]

30. Sonsa-ardjit N, Sakthong P. Reliability and validity of the Thai version of EQ-5D-5L questionnaire on patients with chronic disease. *Chula Med J* 2015;59(5):489-501.

31. Gale NK, Heath G, Cameron E, et al. Using the framework method for the analysis of qualitative data in multi-disciplinary health research. *BMC Med Res Methodol* 2013;13:117. doi: 10.1186/1471-2288-13-117 [published Online First: 2013/09/21]

For peer review only

Figure legend

Figure 1: Study design and recruitment

Group 1: Usual care using warfarin [control] Group 2: Care pathway designated based on stratification of patients using the SAMe-TT_2R_2 score [warfarin plus usual care].

Group 2 care pathways:

Group 2a: SAMe-TT_2R_2 score 0-2 [warfarin plus usual care] NB. Usual care would include management in the anticoagulation clinic, and patient information about AF and need for warfarin by a healthcare professional using the standard warfarin education checklist.

Group 2b: SAMe-TT_2R_2 score >2 group [warfarin plus intensive TREAT education-behavioural intervention as an adjunct to their regular INR monitoring to improve their TTR on warfarin.

Figure 2: Timing of randomization, study visits and follow-up

For peer review only

Appendix

Trial Management Group - TMG of TREATS-AF: Arintaya Phrommintikul, Gregory Lip, G Neil Thomas, Rungroj Krittiyaphong, Wanwarang Wongcharoen, Surakit Nathisuwan, Gemma Slinn, Sukhi Sehmi, Siriluck Gunaparn, Deirdre Lane, Kate Jolly, Sue Jowett, Unchalee Permsuwan, Jonathan Mathers, Wichuda Jiraporncharoen, Chaisiri Angkurawaranon, Anita Slade, Samir Mehta, Antika Wongthanee, Neil Winkle

Trial Steering Committee – TSC: Smonporn Boonyaratavej Songmuang – Chair, Rapeephon Kunjara Na Ayudhya, Lin Yenn-Jiang, Tomorn Thongsri

Data Monitoring and Ethics Committee – DMEC: Tippawan Liabsuetrakul, Kemmawadee Preedalikit, Thoranis Chantrarat,

Participating local investigator list of TREATS-AF;

1. Chiangrai Prachanukroh Hospital: Wattana Wongtheptien, Suparat Wattanasombat, Jutarat Kaewdam, Nongluk Mananusorn, Wuttichai Sawatna

2. Nakornping Hospital: Thanyaluck Chotayaporn, Kultida Lertthanaphol, Busarakam Pothongsunun, Jirarat Chandee, Tananya Prasit, Chotika Ruttanapornnukul, Ketsanee Sripattanatrakul, Vullida Cheingyothakul

For peer review only

3. Maharaj Nakorn Chiang Mai Hospital: Narawudt Prasertwitayakij, Kanokporn Niwatananun, Voratima Silavanich, Mantiwee Nimworapan, Phornwinee Somsap, Tikumporn Pornwisrtsirikul, Praphaphan Daoram, Chayanit Srisongmuang

4. Lampang Hospital: Natrawee Bureekam, Siriporn Intharangsri, Chonlada Kawila

5. Siriraj Hospital: Arjbordin Winigkul, Yodying Kaolawanich, Ajjma Sarapakdi, Usanee Pienpattapong, Olaree Chaiphet

6. Maharat Nakhon Ratchasima Hospital: Bancha Sookananchai, Piti Niyomsiriwanich, Weerapan Wiwatworapan, Ubonwan Sapoo, Suttiwanik Kowitphattana, Ketmanee Sae-Ueng, Arisorn Jirapermpun

7. Srinagarind Hospital and Queen Sirikit Heart Center: Vichai Senthong, Supaporn Onsanit, Siriporn Jantharuechai, Wachiraya Tipboonchu

For peer review only

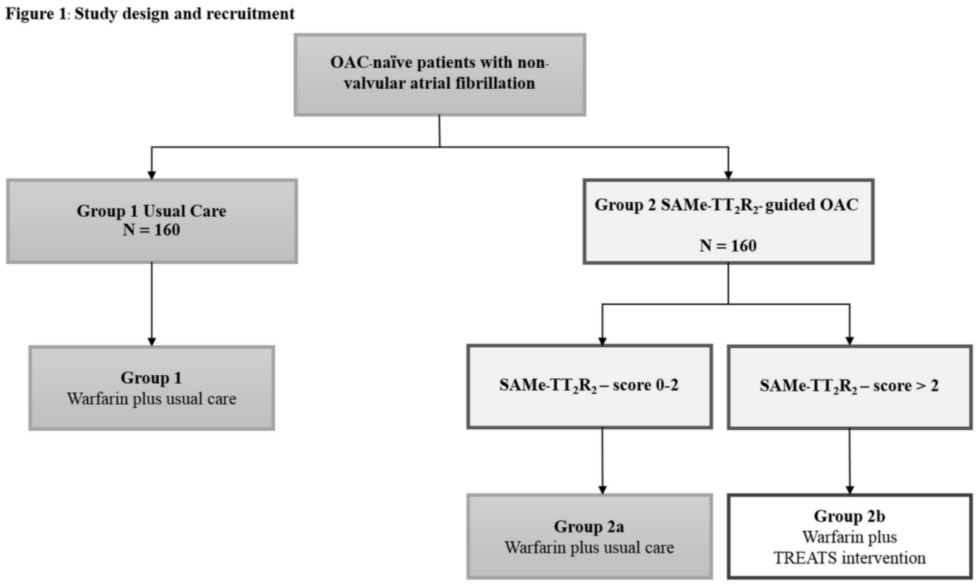

Figure 1: Study design and recruitment
Group 1: Usual care using warfarin [control] Group 2: Care pathway designated based on stratification of patients using the SAMe-TT2R2 score [warfarin plus usual care].
Group 2 care pathways:
Group 2a: SAMe-TT2R2 score 0-2 [warfarin plus usual care] NB. Usual care would include management in the anticoagulation clinic, and patient information about AF and need for warfarin by a healthcare professional using the standard warfarin education checklist.
Group 2b: SAMe-TT2R2 score >2 group [warfarin plus intensive TREAT education-behavioural intervention as an adjunct to their regular INR monitoring to improve their TTR on warfarin.

243x148mm (300 x 300 DPI)

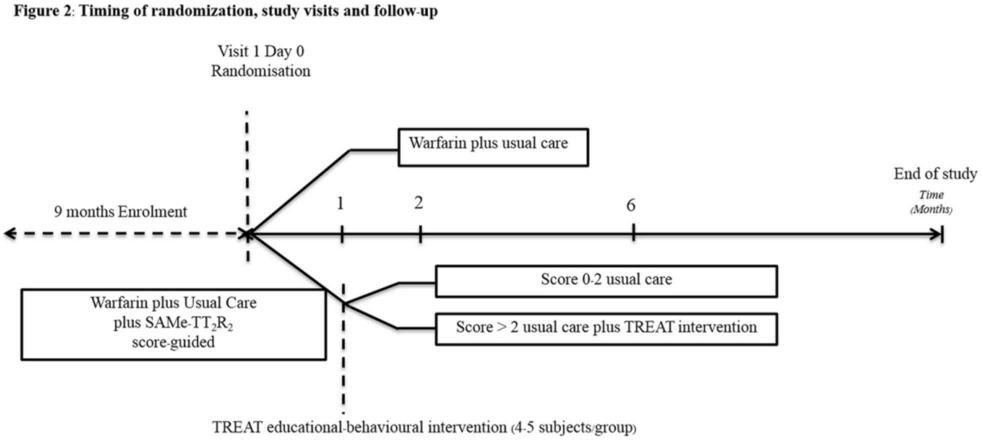

Figure 2: Timing of randomization, study visits and follow-up

129x59mm (300 x 300 DPI)

Appendix

Trial Management Group of TREATS-AF: Arintaya Phrommintikul, Gregory Lip, G Neil Thomas, Rungroj Krittiyaphong, Wanwarang Wongcharoen, Surakit Nathisuwan, Gemma Slinn, Sukhi Sehmi, Siriluck Gunaparn, Deirdre Lane, Catherine B Jolly, Susan Jowett, Unchalee Permsuwan, Jonathan Mathers, Wichuda Jiraporncharoen, Chaisiri Angkurawaranon, Anita Slade, Samir Mehta, Antika Wongthanee, Neil Winkle

Trial Steering Committee – TSC: Smonporn Boonyaratavej Songmuang – Chair, Rapeephon Kunjara Na Ayudhya, Lin Yenn-Jiang, Tomorn Thongsri

Data Monitoring and Ethics Committee – DMEC: Tippawan Liabsuetrakul, Kemmawadee Preedalikit, Thoranis Chantrarat,

Participating local investigator list of TREATS-AF;

1. Chiangrai Prachanukroh Hospital: Wattana Wongtheptien, Suparat Wattanasombat, Jutarat Kaewdam, Nongluk Mananusorn, Wuttichai Sawatna

2. Nakornping Hospital: Thanyaluck Chotayaporn, Kultida Lertthanaphol, Busarakam Pothongsunun, Jirarat Chandee, Tananya Prasit, Chotika Ruttanapornnukul, Ketsanee Sripattanatrakul, Vullida Cheingyothakul

3. Maharaj Nakorn Chiang Mai Hospital: Narawudt Prasertwitayakij, Kanokporn Niwatananun, Voratima Silavanich, Mantiwee Nimworapan, Phornwinee Somsap, Tikumporn Pornwisrtsirikul, Praphaphan Daoram, Chayanit Srisongmuang

4. Lampang Hospital: Natrawee Bureekam, Siriporn Intharangsri, Chonlada Kawila

5. Siriraj Hospital: Arjbordin Winigkul, Yodying Kaolawanich, Ajjma Sarapakdi, Usanee Pienpattapong, Olaree Chaiphet

For peer review only

6. Maharat Nakhon Ratchasima Hospital: Bancha Sookananchai, Piti Niyomsiriwanich, Weerapan Wiwatworapan, Ubonwan Sapoo, Suttiwanik Kowitphattana, Ketmanee Sae-Ueng, Arisorn Jirapermpun

7. Srinagarind Hospital and Queen Sirikit Heart Center: Vichai Senthong, Supaporn Onsanit, Siriporn Jantharuechai, Wachiraya Tipboonchu

For peer review only

CONSORT 2010 checklist of information to include when reporting a randomised trial*

Section/Topic	Item No	Checklist item	Reported on page No
**Title and abstract**			
	1a	Identification as a randomised trial in the title	1
	1b	Structured summary of trial design, methods, results, and conclusions (for specific guidance see CONSORT for abstracts)	2
**Introduction**			
Background and objectives	2a	Scientific background and explanation of rationale	4
	2b	Specific objectives or hypotheses	6
**Methods**			
Trial design	3a	Description of trial design (such as parallel, factorial) including allocation ratio	6
	3b	Important changes to methods after trial commencement (such as eligibility criteria), with reasons	6
Participants	4a	Eligibility criteria for participants	7
	4b	Settings and locations where the data were collected	11
Interventions	5	The interventions for each group with sufficient details to allow replication, including how and when they were actually administered	8
Outcomes	6a	Completely defined pre-specified primary and secondary outcome measures, including how and when they were assessed	9
	6b	Any changes to trial outcomes after the trial commenced, with reasons	9
Sample size	7a	How sample size was determined	-
	7b	When applicable, explanation of any interim analyses and stopping guidelines	-
Randomisation:			
Sequence generation	8a	Method used to generate the random allocation sequence	11
	8b	Type of randomisation; details of any restriction (such as blocking and block size)	7-8
Allocation concealment mechanism	9	Mechanism used to implement the random allocation sequence (such as sequentially numbered containers), describing any steps taken to conceal the sequence until interventions were assigned	7-8
Implementation	10	Who generated the random allocation sequence, who enrolled participants, and who assigned participants to interventions	7-9
Blinding	11a	If done, who was blinded after assignment to interventions (for example, participants, care providers, those	8

For peer review only - http://bmjopen.bmj.com/site/about/guidelines.xhtml

		assessing outcomes) and how	
	11b	If relevant, description of the similarity of interventions	-
Statistical methods	12a	Statistical methods used to compare groups for primary and secondary outcomes	12
	12b	Methods for additional analyses, such as subgroup analyses and adjusted analyses	12
**Results**			
Participant flow (a diagram is strongly recommended)	13a	For each group, the numbers of participants who were randomly assigned, received intended treatment, and were analysed for the primary outcome	13
	13b	For each group, losses and exclusions after randomisation, together with reasons	13
Recruitment	14a	Dates defining the periods of recruitment and follow-up	13
	14b	Why the trial ended or was stopped	-
Baseline data	15	A table showing baseline demographic and clinical characteristics for each group	-
Numbers analysed	16	For each group, number of participants (denominator) included in each analysis and whether the analysis was by original assigned groups	13-14
Outcomes and estimation	17a	For each primary and secondary outcome, results for each group, and the estimated effect size and its precision (such as 95% confidence interval)	13-14
	17b	For binary outcomes, presentation of both absolute and relative effect sizes is recommended	13-14
Ancillary analyses	18	Results of any other analyses performed, including subgroup analyses and adjusted analyses, distinguishing pre-specified from exploratory	13-14
Harms	19	All important harms or unintended effects in each group (for specific guidance see CONSORT for harms)	13
**Discussion**			
Limitations	20	Trial limitations, addressing sources of potential bias, imprecision, and, if relevant, multiplicity of analyses	16
Generalisability	21	Generalisability (external validity, applicability) of the trial findings	-
Interpretation	22	Interpretation consistent with results, balancing benefits and harms, and considering other relevant evidence	-
**Other information**			
Registration	23	Registration number and name of trial registry	6
Protocol	24	Where the full trial protocol can be accessed, if available	6
Funding	25	Sources of funding and other support (such as supply of drugs), role of funders	18

*We strongly recommend reading this statement in conjunction with the CONSORT 2010 Explanation and Elaboration for important clarifications on all the items. If relevant, we also recommend reading CONSORT extensions for cluster randomised trials, non-inferiority and equivalence trials, non-pharmacological treatments, herbal interventions, and pragmatic trials. Additional extensions are forthcoming: for those and for up to date references relevant to this checklist, see www.consort-statement.org.

For peer review only - http://bmjopen.bmj.com/site/about/guidelines.xhtml

BMJ Open

Prospective randomised trial examining the impact of an educational intervention versus usual care on anticoagulation therapy control based on a SAMe-TT2R2 score guided strategy in anticoagulant-naïve Thai patients with atrial fibrillation (TREATS-AF): study protocol

Journal:	*BMJ Open*
Manuscript ID	bmjopen-2021-051987.R1
Article Type:	Protocol
Date Submitted by the Author:	17-Sep-2021
Complete List of Authors:	Phrommintikul, Arintaya; Chiang Mai University Faculty of Medicine, Division of Cardiology, Department of Internal Medicine<br>Nathisuwan, S; Mahidol University Faculty of Pharmacy, Department of Pharmacy<br>Gunaparn, Siriluck; Chiang Mai University Faculty of Medicine, Division of Cardiology, Department of Internal Medicine<br>Krittayaphong, Rungroj; Mahidol University Faculty of Medicine Siriraj Hospital<br>Wongcharoen, Wanwarang; Chiang Mai University Faculty of Medicine, Division of Cardiology, Department of Internal Medicine<br>Sehmi, Sukhi; University of Birmingham Clinical Trials Unit<br>Mehta, Samir; University of Birmingham Clinical Trials Unit<br>Winkles, Neil; University of Birmingham Clinical Trials Unit<br>Brocklehurst, Peter; University of Birmingham Clinical Trials Unit<br>Mathers, Jonathan; University of Birmingham Institute of Applied Health Research<br>Jowett, Sue; University of Birmingham Institute of Applied Health Research, Health Economics Unit<br>Jolly, Kate; University of Birmingham Institute of Applied Health Research<br>Lane, Deirdre; University of Liverpool Faculty of Health and Life Sciences, Cardiovascular Health<br>Thomas, Neil; University of Birmingham Institute of Applied Health Research, Department of Public Health and Epidemiology<br>Lip, Gregory; University of Liverpool, Liverpool Centre for Cardiovascular Science; Aalborg Universitet, Department of Clinical Medicine
**Primary Subject Heading**:	Medical management
Secondary Subject Heading:	Cardiovascular medicine, Health economics, Medical education and training, Qualitative research
Keywords:	CARDIOLOGY, QUALITATIVE RESEARCH, HEALTH ECONOMICS

SCHOLARONE™
Manuscripts

I, the Submitting Author has the right to grant and does grant on behalf of all authors of the Work (as defined in the below author licence), an exclusive licence and/or a non-exclusive licence for contributions from authors who are: i) UK Crown employees; ii) where BMJ has agreed a CC-BY licence shall apply, and/or iii) in accordance with the terms applicable for US Federal Government officers or employees acting as part of their official duties; on a worldwide, perpetual, irrevocable, royalty-free basis to BMJ Publishing Group Ltd ("BMJ") its licensees and where the relevant Journal is co-owned by BMJ to the co-owners of the Journal, to publish the Work in this journal and any other BMJ products and to exploit all rights, as set out in our licence.

The Submitting Author accepts and understands that any supply made under these terms is made by BMJ to the Submitting Author unless you are acting as an employee on behalf of your employer or a postgraduate student of an affiliated institution which is paying any applicable article publishing charge ("APC") for Open Access articles. Where the Submitting Author wishes to make the Work available on an Open Access basis (and intends to pay the relevant APC), the terms of reuse of such Open Access shall be governed by a Creative Commons licence – details of these licences and which Creative Commons licence will apply to this Work are set out in our licence referred to above.

Other than as permitted in any relevant BMJ Author's Self Archiving Policies, I confirm this Work has not been accepted for publication elsewhere, is not being considered for publication elsewhere and does not duplicate material already published. I confirm all authors consent to publication of this Work and authorise the granting of this licence.

For peer review only

Prospective randomised trial examining the impact of an educational intervention versus usual care on anticoagulation therapy control based on a SAMe-TT_2R_2 score guided strategy in anticoagulant-naïve Thai patients with atrial fibrillation (TREATS-AF): study protocol

Arintaya Phrommintikul[1]*, Surakit Nathisuwan[2], Siriluck Gunaparn[1], Rungroj Krittayaphong[3], Wanwarang Wongcharoen[1], Sukhi Sehmi[4], Samir Mehta[4], Neil Winkles[4], Peter Brocklehurst[4], Jonathan Mathers[5], Sue Jowett[6], Kate Jolly[5], Deirdre A. Lane[7], G Neil Thomas[8]*, Gregory Y. H. Lip[9]*; on behalf of the TREATS-AF Study Group**

[*joint senior authors]

[1]Department of Internal Medicine, Faculty of Medicine, Chiang Mai University, Chiang Mai, Thailand. [2]Department of Pharmacy, Faculty of Pharmacy, Mahidol University, Bangkok, Thailand. [3]Faculty of Medicine, Siriraj Hospital, Mahidol University, Bangkok, Thailand. [4]Clinical Trials Unit Birmingham, University of Birmingham, Birmingham, United Kingdom. [5]Institute of Applied Health Research, University of Birmingham, Birmingham, United Kingdom. [6]Health Economics Unit, Institute of Applied Health Research, University of Birmingham Birmingham, United Kingdom. [7]Cardiovascular Health Liverpool, Faculty of Health and Life Sciences, University of Liverpool, Liverpool, United Kingdom. [8]Department of Public Health and Epidemiology, Institute of Applied Health Research, University of Birmingham, Birmingham, United Kingdom. [9]Liverpool Centre for Cardiovascular Science, University of Liverpool and Liverpool Heart & Chest Hospital, Liverpool, United Kingdom; and Department of Clinical Medicine, Aalborg University, Aalborg, Denmark.

**TREATS-AF Study Group

Narawudt Prasertwitayakij, Wattana Wongtheptien, Thanyaluck Chotayaporn, Natrawee Bureekam, Bancha Sookananchai, Vichai Senthong, Antika Wongthanee, Gemma Slinn, Anita Slade

Corresponding authors:

G Neil Thomas: gneilthomas@gmail.com/ g.n.thomas@bham.ac.uk

Gregory Lip: Gregory.Lip@liverpool.ac.uk

For peer review only

Running head: Educational intervention for anticoagulation management

Word count: Abstract: 289 words; Main Text: 3,914 words

Tables: 2 Figures: 2

For peer review only

Abstract

Introduction: The burden of atrial fibrillation (AF) in Thailand is high and associated with increased morbidity, mortality and healthcare costs. Vitamin-K antagonists (VKA, e.g. warfarin), commonly used for stroke prevention in AF patients in Thailand, are effective but are often sub-optimally controlled. We aim to evaluate the impact of a SAMe-TT_2R_2 score guided strategy and educational intervention compared to usual care on anticoagulation control expressed by the time in therapeutic range (TTR) at 12-months, in anticoagulant-naïve Thai patients with AF.

Methods and analysis: Multi-centre, open-label, parallel-group, randomised controlled trial conducted in Thailand among adult patients (age ≥ 18 years) with AF who are anticoagulant-naïve. Patients will be randomised to one of two groups; a SAMe-TT_2R_2 score guided strategy with educational intervention and usual care vs. usual care alone. The planned follow-up period is 12 months. The primary outcome is time in therapeutic range (TTR) at 12 months.Secondary outcomes include: 1) TTR at 6 months; 2) thromboembolic and bleeding events at 12 months; 3) composite major adverse cardiovascular events (MACE) at 12 months; 4) change in patients' knowledge of AF between baseline and 6 months and 12 months; 5) cost-effectiveness; 6) quality-of-life at baseline, 6 and 12 months using EQ-5D-5L (Thai version); and 7) patient satisfaction/perceptions of the TREAT intervention. An embedded qualitative study will assess patient perceptions of the TREAT intervention.

Ethics and dissemination: The study has been approved by the Ethical Review Committee, Ministry of Public Health of Thailand and registered in The Thai Clinical Trials Registry (TCTR20180711003). The results of this trial will be submitted for publication in a peer-reviewed journal. Participants will be informed via a link to a preview of the publication. A lay summary will also be provided to all participants prior to publication.

Keywords: anticoagulants, atrial fibrillation, SAMe-TT2R2 score, warfarin

For peer review only

Strengths and limitations of this study

- Warfarin remains the default therapy for stroke prevention in atrial fibrillation (AF) in many countries, but is often sub-optimally managed.
- A one-time structured educational-behavioural intervention (TREAT intervention) among those predicted to be less likely to achieve optimal control, may be a simple and cost-effective adjunct management strategy which could help improve time in therapeutic range**.**
- This prospective individually-randomised-controlled trial (RCT) in anticoagulant-naïve patients with AF aims to assess whether the application of a SAMe-TT_2R_2 score-guided strategy and TREAT intervention plus usual care could improve anticoagulation control with warfarin.
- The study design has a multidisciplinary team approach, and the efficacy, safety, patients' satisfaction as well as cost-effectiveness will be assessed.
- One challenge of the study – if implemented - is to standardise the quality of usual care and TREAT intervention among centers.

For peer review only

Introduction

Atrial fibrillation (AF) is associated with decreased quality of life, and increased mortality and morbidity from stroke/thromboembolism.[1 2] Incidence of AF has been increasing in developing countries including Thailand, partly due to the rapid change of population dynamics towards an ageing society.[3 4] The hospitalisation rate for Thai AF patients was high (15.5 per 100,000 person-years), with a high mortality (44.0% after 72-month follow-up (average 46 months)).[5] The high burden of AF in Thailand is also associated with similarly high healthcare costs[6].

Appropriate oral anticoagulation, either with a vitamin-K antagonist (VKA, e.g. warfarin) or non-VKA oral anticoagulants (NOAC), is essential for effective stroke prevention in AF patients with one or more stroke risk factors.[7] Despite having various advantages over VKA, prohibitive costs means access to NOACs are generally limited for low-to-middle income countries such that VKAs are widely used globally[8 9]. In Thailand, warfarin is the default therapy for stroke prevention in AF, but is often sub-optimally managed due to the many inherent limitations associated with this drug, including diet-, drug-, and alcohol-interactions and difficulties with anticoagulation monitoring. Recent data from retrospective cohort studies, along with a nationwide registry, suggested that the average TTR among AF patients in Thailand ranged from 50 to 55%. [10-13] Those with sub-optimal anticoagulation control, TTR<65%, had a 2 to 3-fold increased risk of stroke, major bleeding, and death compared to those with TTR ≥65%.[12] As a result, strategies to improve TTR control are clearly needed and would likely have a major impact on preventing these adverse events.

TTR is influenced by numerous modifiable and non-modifiable factors, but the more common clinical factors have been used to formulate the SAMe-TT_2R_2 score (Table 1).[14]

Table 1. Components of the SAMe-TT$_2$R$_2$ score

**Components**		**Points**
**S**	Sex (female)	1
**A**	Age (<60 years)	1
**Me**	Medical history*	1
**T**	Treatment (interacting drug e.g., amiodarone)	1
**T**	Tobacco use (within 2 years)	2
**R**	Race (non-Caucasian)	2
Maximum total		8
* More than two of the following: hypertension, diabetes mellitus, coronary artery disease/myocardial infarction, peripheral atrial disease, congestive heart failure, previous stroke, pulmonary disease, hepatic or renal disease		

SAMe-TT2R2 scores 0-2; the patients with AF likely to achieve and maintain optimal TTR and SAMe-TT2R2 > 2; the patients with AF likely poorer responders.

Several observational studies including those conducted in Asian populations have demonstrated that a SAMe-TT$_2$R$_2$ value of >2 is predictive of poor TTR, all-cause mortality and the composite end-point of thromboembolic events, major bleeding and mortality.[13 15-17] By identifying those who are at risk of poor TTR, targeted efforts to improve TTR can be effectively provided to the vulnerable group. If TTR can be improved to >65%, VKAs can provide comparable efficacy and safety to that seen with NOACs.[18 19] Previous studies have demonstrated that many patients with AF possess little knowledge about AF and do not understand the risks/benefits of OAC,[20-25] and this may contribute to poor INR control.

We have previously shown that an educational-behavioural intervention (TREAT, ISRCTN93952605) significantly improved TTR 6-months after warfarin initiation compared to usual care alone (78.5% vs. 66.7% respectively; p=0.01).[26-28] Thus, a one-time structured educational-behavioural intervention among those predicted to be less likely to achieve good INR control, may be a simple and cost-effective adjunct management strategy which could help improve individual TTR. Increased patient

For peer review only

understanding of disease and treatment, and a reduction of adverse events, may improve quality of life.

We therefore conducted a prospective individually-randomised-controlled trial (RCT) in anticoagulant-naïve Thai patients with AF to assess whether the application of a SAMe-TT_2R_2 score-guided strategy and TREAT intervention[26-28] plus usual care could improve anticoagulation control with warfarin (measured by percentage TTR) at 12 months (primary outcome) compared with usual care alone.

Objectives

The primary objective of this study is to evaluate the impact of a SAMe-TT_2R_2 score guided strategy and educational intervention (plus usual care) compared to usual care alone, on patient's anticoagulation control with warfarin, as measured by the time in therapeutic range (TTR) at 12 months, in anticoagulant-naïve Thai patients with AF. As secondary objectives, we aim to evaluate the impact of this intervention on patient knowledge, TTR at 6 months, thromboembolic and bleeding events and the composite major adverse cardiovascular events (MACE). We will also perform analysis to evaluate the cost-effectiveness of the intervention and investigate patients' satisfaction and acceptance of the TREAT intervention using a brief questionnaire and further in a qualitative sub-study.

Methods

Study design & setting

This is a multi-centre, open-label, paralleled-group, RCT conducted among adult Thai patients (age ≥18 years) with AF who are anticoagulant-naïve. Patients who are eligible for stroke prevention according to the ESC Guidelines for the diagnosis and management of atrial fibrillation[1] (men with CHA_2DS_2VASc score ≥1; women with CHA_2DS_2VASc score ≥2) will be randomised to one of two groups, either a SAMe-TT_2R_2 score guided strategy (see Table 1) and educational intervention plus usual care vs. usual care alone. The planned follow-up period is 12 months. Study sites are seven

For peer review only

public, hospitals across six provinces (Bangkok, Chiang Mai, Chiang Rai, Lampang, Khon Kaen and Nakhon Ratchasima) in Thailand. To promote generalisability of our findings, these seven hospitals are a combination of three university hospitals (Maharaj Nakorn Chiang Mai Hospital, Siriraj Hospital, and Queen Sirikit Heart Center of the Northeast) and four secondary/tertiary care hospitals (Maharat Nakhon Ratchasima Hospital, Chiang Rai Prachanukroh Hospital, Nakornping Hospital, and Lampang Hospital). The study sites and investigators are listed in the supplementary file. The study protocol was approved by the Institutional Ethical Committee of each study site and by the IRB of the Ministry of Public Health, the Royal Government of Thailand (Central Research Committee (CREC) No. COA-CREC 007/2020) and registered with the Thai Clinical Trials Registry reference number: TCTR20180711003).

Participants

Newly diagnosed adult AF patients identified and referred from cardiology, internal medicine, family medicine and general clinics at the hospitals and referrals from primary care will be eligible for enrolment into the study.

Eligibility Criteria

Patient must meet all of the following inclusion criteria to be eligible for randomisation:

(1) ≥18 years of age

(2) Newly diagnosed non-valvular AF patients

(3) ECG-documented AF

(4) Warfarin-eligible (men with CHA_2DS_2VASc score ≥1; women with CHA_2DS_2VASc score ≥2)

(5) Warfarin-naïve (no treatment with anticoagulation within the past 12 months, treatment may have started within the prior 28 days from randomisation

(6) Able to comply with scheduled visits, treatment plan and laboratory tests

(7) Able to give informed consent and comply with study protocol (with support of a carer)

Patients will be excluded from participation if they present with any of the following:

(1) Any contraindication to oral anticoagulation

(2) Prosthetic cardiac valve or significant valvular heart disease with an indication for heart surgery

For peer review only

(3) Likelihood of intermittent or permanent discontinuation of warfarin during follow-up (e.g., Major surgery or post-AF ablation)

(4) Known active malignancy with a life expectancy less than 5 years

(5) Diagnosed significant cognitive impairment preventing provision of informed consent and/or able to comply with the study protocol

(6) Any disease likely to cause death within 12 months

The study was originally designed to enroll the patients within 5 days of warfarin treatment, however the average referral time was 14 days. The trial steering committee agreed to extend the duration between starting warfarin to randomization to 28 days to improve recruitment rate, external validity and generalizability of the trials. The amended study protocol was approved by the Institutional Ethical Committee of each study site and by the IRB of the Ministry of Public Health, the Royal Government of Thailand and updated in the Thai Clinical Trials Registry."

Randomisation

After participant eligibility has been confirmed and informed consent has been provided, the participant will be randomised into the trial. Randomisation will be done using a web-based platform with blinded allocation. Randomisation will be stratified based on centre, sex (male or female) and baseline SAMe-TT_2R_2 score (0-2, 3-5, and 6-8). Participants will be randomised at the level of the individual in a 1:1 ratio to either the SAMe-TT_2R_2 score guided strategy and educational intervention plus usual care (intervention group) or usual care alone. (Figure 1 and 2) Group 1 is the usual care group using warfarin (control group). For Group 2, the patients are further divided into two groups. Group 2a are patients with a SAMe-TT_2R_2 score 0-2 who will receive warfarin plus usual care. Group 2b are patients with SAMe-TT_2R_2 score >2 group who will receive warfarin plus the TREAT education-behavioural intervention as an adjunct to their regular INR monitoring, to improve their TTR.

Trial intervention

As described previously, the TREAT intervention is a patient-centred intervention for patients with AF co-developed through the integration of theoretical and clinical frameworks, and patient feedback.[26 27] This behaviour-change intervention package consists of an educational booklet, diary,

worksheet and a DVD for reinforcement. The educational booklet covers AF causes and consequences, warfarin and its metabolism, stroke risk and risk of bleeding on treatment and lifestyle changes (diet, alcohol, lifestyle change). The patient DVD contains health professionals and 'expert patient' narratives that covers AF causes, consequences, side effects, treatment options, warfarin, INR monitoring, lifestyle changes, along with common psychological and physical barriers to anticoagulation that patients may experience. The patient worksheets include a simple self-calculation of stroke risk (CHA_2DS_2-VASc score), personal barriers to uptake of warfarin, and discussion of personal goals for lifestyle changes. In addition, a self-monitoring diary including diet, alcohol intake (in units), warfarin regimen and INR results is also provided.

To overcome the language barrier of the general Thai public toward English, the TREAT intervention was culturally adapted to fit the local context and then translated. Forward and backward translation by two bilingual experts was performed. A test run of the Thai-version of the TREAT intervention was conducted among AF patients with a range of educational ability (Thai literacy level is 94%) and age levels. Patient feedback was incorporated to improve the usability of the Thai-version TREAT intervention.

A training session was conducted to prepare all study site coordinators (pharmacists and nurses) on how to deliver the intervention effectively. To avoid contamination, those who provide the TREAT intervention and those who provide usual care will be two different groups of pharmacists and nurses. The intervention will be delivered within 4-weeks of initiating warfarin. Intervention fidelity will be ensured by direct observation of random sessions at each centre by the trainer. Patients in the usual care group will receive training on the OAC management from the anticoagulation clinic, and patient information about AF and need for warfarin by a healthcare professional using the standard warfarin education checklist.

Patient and Public Involvement

Patients or the public were not involved in the design, or conduct, or reporting, or dissemination plans of our research. The patients involved in the development of Thai-version of the TREAT intervention.

For peer review only

Outcome measures

The primary outcome is time in therapeutic range (TTR) at 12 months. Secondary outcomes include 1) TTR at 6-months; 2) thromboembolic and bleeding events from at 12 months; 3) composite major adverse cardiovascular events (MACE) outcome of non-fatal MI, non-fatal stroke and cardiovascular death and all-cause mortality, in an exploratory analysis (combined and individually) at 12 months; 4) change in patients' knowledge of AF from baseline to 6 months and 12 months; 5) cost-effectiveness; 6) quality-of-life at baseline, 6 and 12 months assessed using EQ-5D-5L (Thai version)[29 30]; and 7) patient satisfaction/perceptions of the TREAT intervention. An embedded qualitative study will assess patient perceptions of the TREAT intervention.

Study procedures and schedules of assessment

Baseline SAMe-TT_2R_2 score will be calculated (Table 1). All patients will receive warfarin, dose-adjusted to achieve a target INR of 2.0 to 3.0. INR monitoring will be undertaken at routine intervals (as would occur in usual care) and will be recorded over a 12-month period. Patients will be randomised as described in Figure 1, either to Group 1 (usual care) or Group 2 (intervention arm), for which the trial is powered. Group 2 is a directed management group based on a: patients with AF likelihood of achieving and maintaining optimal TTR (SAMe-TT_2R_2 scores 0-2; Group 2a) and those likely to achieve poor TTR (SAMe-TT_2R_2 scores >2, Group 2b). From our prior data, approximately 15%-20% will likely enter Group 2a. The primary and secondary outcomes between Group 1 and Group 2 (together) will be compared. The schedule of assessment is presented in Table 2. INR monitoring is independent of the intervention (i.e. naturalistic) as we recognise that the improvement of TTR may be a result of more frequent monitoring. Patients in all groups will have their INR monitored as they would if they were not in the trial (by anticoagulation services which are separate from the trial and who are blinded to treatment allocation).

For peer review only

Table 2. Study visits schedule

CLINICAL ASSESSMENT:

- A. Demographic information: age, sex, ethnicity, education level, height (cm), weight (kg), body mass index, smoking status, alcohol use.
- B. Medical history: history of arterial hypertension, heart failure, diabetes mellitus, coronary artery disease (myocardial infarction, PCI, CABG), peripheral artery disease, stroke, transient ischemic attack, systemic embolism, COPD, chronic kidney disease, chronic liver disease, thyroid disease, bleeding event
- C. Physical exam, blood pressure, heart rate, EHRA and NYHA class
- D. Concomitant medical therapy

OAC THERAPY EDUCATION: ALL PATIENT-a standard OAC education session conducted by a healthcare professional about the clinical significance and management of OAC therapy

QUESTIONNAIRES: Atrial fibrillation knowledge questionnaires, patient satisfaction (Likert scale) and EQ-5D-5L (Thai version)

HEALTH ECONOMICS ASESSMENT: EQ-5D-5L (Thai version), AF, warfarin and clinical event-related healthcare resource use, intervention costs

FOLLOW-UP OF CLINICAL EVENTS: TTR; composite of thromboembolism, major bleeding, all-cause death (in all patients).

	**BASELINE**	**M1±7D**	**M2±7D**	**M6±14D**	**M12±14D**
**CLINICAL ASSESSMENT**	**a,b,c,d**	**c**	**c**	**c**	**c,d**
**ECG**	x				
**INR†**	x	x	x	x	x
**STANDARD OAC THERAPYEDUCATION**	x				
**TREAT intervention**		x			
**QUESTIONNAIRES**	x			x	x
**QUALITATIVE ASSESSMENT**	x‡			x	
**HEALTH ECONOMIC ASSESSMENT**	x			x	x
**FOLLOW-UP OF CLINICAL EVENTS**				x	x

†INR MONITTORING IS MANDATORY during the 12 month follow-up period for all patients, with frequency as per usual care conducted by anticoagulation clinics

‡Baseline interviews of patients will take place within 4 weeks of receiving the intervention

For peer review only

Data collection and management

Medical records of the participating hospitals serve as the source data including clinical information and laboratory values which are accessible and maintained in accordance to national standards and regulations. Outcome data will be extracted from participant's clinical notes and laboratory reports into an electronic CRF. For long term storage, clinical data will be electronically archived at Faculty of Medicine, Chiang Mai University, Thailand as per the regulatory requirements for Good Clinical Practice and in line with the Medical Research Council (MRC) requirements and local policies.

The questionnaires will be completed by the patient. For those with literacy problems, the questionnaires will be read out to the participant, the participant will respond to each question and their responses will be recorded by the staff member administering the questionnaires.

Trial data will be captured in a REDCAP, secure SQL server database. An independent Data Monitoring Committee (DMC), including both local and international experts, has been appointed and convened. Interim analyses of major outcome measures and safety data will be conducted and provided in strict confidence to the DMC. Any decision to stop the trial early will be based on the balance of efficacy and safety.

Sample size and power calculations

Most recent studies suggest that the mean TTR in Thai patients receiving warfarin is in the range of 50%-55%.[11 12] Since an improvement of 10% in the mean TTR is likely to be clinically meaningful, we assume a 10% difference in 12-month TTR with 90% power and 5% significance level for our sample size calculation and using a standard deviation of "26" as observed in a previous Thai study to determine the sample size estimates. Hence to detect a 10% mean difference in TTR at 12 months, using two-sided test, sample size for randomisation in a 1:1 ratio would be 288 or 144 per arm. Assuming and adjusting for a 10% attrition/loss to follow-up rate, a total sample size of 320 patients or 160 per arm is needed.

For peer review only

Statistical analysis

Continuous variables will be presented as mean (SD), or median (interquartile range, IQR), as appropriate; categorical variables will be reported as counts with percentages. Descriptive statistics will be presented for baseline demographic and clinical information. For all major outcomes, summary statistics and differences between groups (e.g. mean differences, relative risks) will be presented, with confidence intervals and p-values from two-sided tests also given.

All analyses will be based on the intention to treat (ITT) principle. The primary comparison groups will be composed of those randomised to Group 1, usual care versus those randomised to Group 2, the SAMe-TT_2R_2 guided strategy with the TREAT intervention (plus usual care). All outcomes will be adjusted for the stratification variables (centre, sex and baseline SAMe-TT_2R_2 score) where possible. Two tailed p-values <0.05 will be considered statistically significant. No adjustment for multiple comparisons will be made.

Sub-group analysis

Sub-group analyses will be conducted for the stratification variable sex only. All other stratification variables will be used only to ensure that the two groups are balanced in terms of these important variables. Sub-group analyses will be limited to the primary outcome only. Tests for statistical heterogeneity (e.g. by including the treatment group by subgroup interaction parameter in the regression model) will be performed prior to any examination of effect estimates within subgroups. The results of sub-group analyses will be treated with caution and will be used for the purposes of hypothesis generation only.

Missing Data and Sensitivity Analyses

Missing and ambiguous data will be queried using a data clarification system, and will focus on data required for trial outcome analysis and safety reporting. Every attempt will be made to collect full follow-up data on all study participants. A sensitivity analysis will be performed on the primary outcome measure only to examine the possible impact of any missing data for the primary outcome. This will include the use of multiple imputation using chained equations. Further sensitivity analysis will also be done if a substantial number of participants do not comply with randomised allocated

For peer review only

group and so a per-protocol analysis may also be conducted. Any sensitivity analyses will not, irrespective of their differences, supplant the planned primary analyses.

Interim Analysis

Interim analyses of safety and efficacy for presentation to the independent DMC will take place during the study. The committee will meet prior to study commencement to agree the manner and timing of such analyses. The analysis of the primary and major secondary outcomes and full assessment of safety (adverse events and serious adverse events) will be performed at annual intervals. Criteria for stopping or modifying the study based on this information will be ratified by the DMC.

Planned Final Analyses

The final analysis for the study will occur once the last randomised participant has completed the 12 month follow-up and any corresponding outcome data has been entered onto the study database and validated as being ready for analysis.

Withdrawal

Patients may withdraw consent from the study at any time. Patients may also withdraw from trial treatment but continue with study follow-up and data collection as per the protocol. If the withdrawal is initiated by a health care professional, full details for the reason for withdrawal will be recorded on the case report forms. In all other cases, a simple statement reflecting the patient's preference will be noted. The patients who are switched to NOACs will be followed for clinical events. The INR data will be collected to the end of warfarin treatment for the TTR calculation and analysis as intention to treat analysis.

Analysis of outcome measures

Primary outcome

The primary outcome is the TTR at 12 months and differences in TTR between the intervention and usual care groups will be examined using a linear regression model. Results will be presented as mean difference and 95% confidence interval. A key assumption for this analysis is that the data will be normally distributed; this assumption will be examined visually based on the residuals from the fitted model. If data is deemed not to be normally distributed, then the Mann Whitney U test will be used for analysis.

For peer review only

Secondary outcomes

The secondary endpoints for the trial includes continuous, categorical and time-to-event data items. Any secondary endpoints that are continuous in nature will be analysed in the same way as described for the primary outcome. For dichotomous secondary endpoints, the proportion of participants and percentages will be compared between arms using a log-binomial model. Relative risks and 95% confidence intervals will be calculated. For any time-to-event outcomes these will be compared between treatment arms by using survival analysis methods. Cox proportional hazards model will be used and treatment effects will be expressed as hazard ratios with 95% confidence intervals. Kaplan-Meier survival curves will be constructed for visual presentation of time-to-event comparisons.

Qualitative study

Qualitative data will be obtained directly from patient interviews and interviews with healthcare professionals delivering the intervention as part of an embedded qualitative sub-study. A purposive sample of 10-15 participants from the intervention arm will take part in one-to-one semi-structured interviews with a qualitative researcher, at two time-points (within 4 weeks of the intervention and at 6-months follow-up). Interviews will explore participants' experience of AF; of receiving the TREAT intervention; their perspectives on the utility of different interventions components; as well as behavioural modifications resulting from the intervention. Staff delivering the intervention at each study site will also be interviewed to understand their perceptions of the TREAT intervention and their experience of delivering it to patients. A thematic analysis of interview content will be informed by the Framework analytic approach. Audio-recorded interviews will be translated and transcribed into English in order to facilitate team-based analysis by Thai and English research team members[31].

Health economics analysis

The economic evaluation will determine the cost-effectiveness of the intensive educational intervention versus usual care. The evaluation will take the form of an incremental cost-utility analysis to estimate cost per quality adjusted life year (QALY) over 12 months follow up from a

For peer review only

Thailand health service perspective. If differences in TTR are achieved in the trial, a longer-term Markov model-based cost per QALY analysis will be also undertaken.

Data collection

Data will be collected on health care utilisation required for the intervention, warfarin therapy and monitoring, and health care visits, hospitalisations, investigations, and medication related to thromboembolism, bleeding and management of AF. Information will be collected at the 6 and 12-month patient visits from trial CRFs and hospital records. Additional costings, such as those associated with transportation to the hospital, will be collected from the patients. The EQ-5D-5L (Thai version) questionnaire will be administered to participants at baseline, 6, and 12 months to estimate QALYs.

Data analysis

Unit costs from standard Thai sources will be applied to all health care resource use items, and mean resource use (for each category of health care usage) and mean total costs will be calculated for both trial arms. The current Thailand value set will be applied to patient EQ-5D-5L responses to obtain utility scores and QALYs will be calculated using the area under the curve approach. Multiple imputation will be used to impute all missing values for the EQ-5D-5L and total cost estimates for non-responders. As cost and QALY data is likely to have a skewed distribution, a non-parametric comparison of means (using bootstrapping) will be undertaken. A cost-consequence analysis will initially be reported, describing all the important results relating to resource use, costs and consequences. Incremental cost-effectiveness and cost-utility analyses will then be undertaken to estimate the incremental cost per QALY gained at 12 months, with adjustment for baseline covariates. The robustness of the results will be explored using sensitivity analysis. Cost-effectiveness acceptability curves (CEACs) will also be produced to reflect the probability the intervention will be cost effective at different cost per QALY willingness to pay thresholds.

Model-based analysis

If the trial data shows a benefit in TTR due to the intervention, trial results will be extrapolated using a Markov model-based cost per QALY. This model will take into account the long-term impact of

For peer review only

anticoagulant control on thromboembolism and bleeding over the patient's lifetime, with costs and outcomes discounted at 3%. The model structure will be informed by previously published modelling studies and expert opinion. Extensive deterministic sensitivity analysis using pre-defined ranges will be undertaken to explore which model parameters have most impact on cost-effectiveness. Parameter estimates will be incorporated into the model as distributions to allow for a probabilistic sensitivity analysis, with CEACs constructed to assess the probability of cost effectiveness at different cost per QALY willingness to pay thresholds.

Trial status

The study protocol version 2.1 date 19 AUG 2019 was approved by the Institutional Ethical Committee of each study site and by the IRB of the Ministry of Public Health, the Royal Government of Thailand (Central Research Committee (CREC) No. COA-CREC 007/2020) and the study protocol had amended and approved at version 3.0 date 9 JUN 2020. The recruitment of participants started in 31st January 2020. Last patient recruitment is expected in May 2021. Data collection is expected to be completed in December 2021.

Dissemination

Results of this trial will be submitted for publication in a peer-reviewed journal. The manuscript will be prepared by the principal investigators or their delegates and authorship will be determined by the trial publication policy. Participants will be informed of the outcome of the trial via a link to a preview of the publication. A lay summary will also be provided via email or posted to participants prior to publication.

For peer review only

Acknowledgments

We acknowledge the assistance and facilities provided by the Medical Research Council (MRC/ Birmingham Clinical Research Facility.

Funding information:

This study is supported by the Newton Fund through the collaboration of the Medical Research Council (MRC), the United Kingdom, the grant reference number is MR/R020892/1 and the Thailand Research Fund (TRF), Thailand, and the grant reference number is DBG6180009.

Authors' contributions:

AP, NT, GL initiated the study. AP is the principal investigator of RCT. SG is the clinical trial manager. AP,NT,SN and GL led the protocol development with contribution from SN, SG, RK, WW, SS, SM, NW, PB, JM, SJ, KJ, DL. SN, WW prepared educational content including educational booklet, diary, worksheet and a DVD. SN conducted TREAT intervention training for pharmacists and monitored during the trial. AP, WW, RK conduct intervention activities together with research assistants. All authors read, contributed to and approved the final version of the manuscript.

Conflict of interest statement:

All other authors have no actual or potential conflict of interest capable of influencing judgment on the part of any author on this work.

AP: reports speaker fees from Bayer, Pfizer, Boehringer Ingelheim, and Daiichi-Sankyo outside the submitted work.

WW: reports speaker fees from Bayer, Pfizer, Boehringer Ingelheim, and Daiichi-Sankyo outside the submitted work.

DL: reports investigator-initiated educational grants from Bristol-Myers Squibb (BMS), speaker fees for Boehringer Ingelheim, Bayer, and BMS/Pfizer and consultancy for BMS/Pfizer, Boehringer Ingelheim, and Daiichi-Sankyo, all outside of the submitted work.

GL: reports consultancy and speaker fees for BMS/Pfizer, Boehringer Ingelheim, and Daiichi-Sankyo. No fees are received personally.

Data sharing: Data are available upon reasonable request.

For peer review only

Reference

1. Hindricks G, Potpara T, Dagres N, et al. 2020 ESC Guidelines for the diagnosis and management of atrial fibrillation developed in collaboration with the European Association of Cardio-Thoracic Surgery (EACTS). *Eur Heart J* 2020 doi: 10.1093/eurheartj/ehaa612 [published Online First: 2020/08/30]

2. Lip GYH, Banerjee A, Boriani G, et al. Antithrombotic Therapy for Atrial Fibrillation: CHEST Guideline and Expert Panel Report. *Chest* 2018;154(5):1121-201. doi: 10.1016/j.chest.2018.07.040 [published Online First: 2018/08/26]

3. Chugh SS, Havmoeller R, Narayanan K, et al. Worldwide epidemiology of atrial fibrillation: a Global Burden of Disease 2010 Study. *Circulation* 2014;129(8):837-47. doi: 10.1161/CIRCULATIONAHA.113.005119 [published Online First: 2013/12/19]

4. Phrommintikul A, Detnuntarat P, Prasertwitayakij N, et al. Prevalence of atrial fibrillation in Thai elderly. *J Geriatr Cardiol* 2016;13(3):270-3. doi: 10.11909/j.issn.1671-5411.2016.03.002 [published Online First: 2016/04/23]

5. Apiyasawat S, Tangcharoen T, Wisaratapong T, et al. CHA(2)DS(2)-VASc scores predict mortality after hospitalization for atrial fibrillation. *Int J Cardiol* 2015;185:293-6. doi: 10.1016/j.ijcard.2015.03.180 [published Online First: 2015/03/31]

6. Ng SS, Nathisuwan S, Phrommintikul A, et al. Cost-effectiveness of warfarin care bundles and novel oral anticoagulants for stroke prevention in patients with atrial fibrillation in Thailand. *Thromb Res* 2020;185:63-71. doi: 10.1016/j.thromres.2019.11.012 [published Online First: 2019/11/27]

7. Kirchhof P, Benussi S, Kotecha D, et al. 2016 ESC Guidelines for the management of atrial fibrillation developed in collaboration with EACTS. *Europace : European pacing, arrhythmias, and cardiac electrophysiology : journal of the working groups on cardiac pacing, arrhythmias, and cardiac cellular electrophysiology of the European Society of Cardiology* 2016;18(11):1609-78. doi: 10.1093/europace/euw295 [published Online First: 2016/11/04]

For peer review only

8. Krittayaphong R, Phrommintikul A, Ngamjanyaporn P, et al. Rate of anticoagulant use, and factors associated with not prescribing anticoagulant in older Thai adults with non-valvular atrial fibrillation: A multicenter registry. *J Geriatr Cardiol* 2019;16(3):242-50. doi: 10.11909/j.issn.1671-5411.2019.03.004 [published Online First: 2019/05/14]

9. Tse HF, Wang YJ, Ahmed Ai-Abdullah M, et al. Stroke prevention in atrial fibrillation--an Asian stroke perspective. *Heart Rhythm* 2013;10(7):1082-8. doi: 10.1016/j.hrthm.2013.03.017 [published Online First: 2013/03/19]

10. Saokaew S, Sapoo U, Nathisuwan S, et al. Anticoagulation control of pharmacist-managed collaborative care versus usual care in Thailand. *Int J Clin Pharm* 2012;34(1):105-12. doi: 10.1007/s11096-011-9597-8 [published Online First: 2011/12/29]

11. Priksri W, Rattanavipanon W, Saejear W, et al. Incidence, risk factors, and outcomes of warfarin-associated major bleeding in Thai population. *Pharmacoepidemiol Drug Saf* 2019;28(7):942-50. doi: 10.1002/pds.4781 [published Online First: 2019/05/09]

12. Krittayaphong R, Chantrarat T, Rojjarekampai R, et al. Poor Time in Therapeutic Range Control is Associated with Adverse Clinical Outcomes in Patients with Non-Valvular Atrial Fibrillation: A Report from the Nationwide COOL-AF Registry. *J Clin Med* 2020;9(6) doi: 10.3390/jcm9061698 [published Online First: 2020/06/06]

13. Zulkifly H, Lip GYH, Lane DA. Use of the SAMe-TT2R2 score to predict anticoagulation control in atrial fibrillation and venous thromboembolism patients receiving vitamin K antagonists: A review. *Heart Rhythm* 2018;15(4):615-23. doi: 10.1016/j.hrthm.2017.11.026 [published Online First: 2017/11/29]

14. Apostolakis S, Sullivan RM, Olshansky B, et al. Factors affecting quality of anticoagulation control among patients with atrial fibrillation on warfarin: the SAMe-TT(2)R(2) score. *Chest* 2013;144(5):1555-63. doi: 10.1378/chest.13-0054 [published Online First: 2013/05/15]

15. Lip GYH, Haguenoer K, Saint-Etienne C, et al. Relationship of the SAMe-TT(2)R(2) score to poor-quality anticoagulation, stroke, clinically relevant bleeding, and mortality in patients with atrial fibrillation. *Chest* 2014;146(3):719-26. doi: 10.1378/chest.13-2976 [published Online First: 2014/04/12]

For peer review only

16. Bernaitis N, Ching CK, Chen L, et al. The Sex, Age, Medical History, Treatment, Tobacco Use, Race Risk (SAMe TT2R2) Score Predicts Warfarin Control in a Singaporean Population. *J Stroke Cerebrovasc Dis* 2017;26(1):64-69. doi: 10.1016/j.jstrokecerebrovasdis.2016.08.030 [published Online First: 2016/09/28]

17. Chan PH, Hai JJ, Chan EW, et al. Use of the SAMe-TT2R2 Score to Predict Good Anticoagulation Control with Warfarin in Chinese Patients with Atrial Fibrillation: Relationship to Ischemic Stroke Incidence. *PLoS One* 2016;11(3):e0150674. doi: 10.1371/journal.pone.0150674 [published Online First: 2016/03/25]

18. Sjogren V, Grzymala-Lubanski B, Renlund H, et al. Safety and efficacy of well managed warfarin. A report from the Swedish quality register Auricula. *Thromb Haemost* 2015;113(6):1370-7. doi: 10.1160/TH14-10-0859 [published Online First: 2015/02/27]

19. Bjorck F, Renlund H, Lip GY, et al. Outcomes in a Warfarin-Treated Population With Atrial Fibrillation. *JAMA Cardiol* 2016;1(2):172-80. doi: 10.1001/jamacardio.2016.0199 [published Online First: 2016/07/22]

20. Lip GY, Kamath S, Jafri M, et al. Ethnic differences in patient perceptions of atrial fibrillation and anticoagulation therapy: the West Birmingham Atrial Fibrillation Project. *Stroke* 2002;33(1):238-42. doi: 10.1161/hs0102.101817 [published Online First: 2002/01/10]

21. Lane DA, Aguinaga L, Blomstrom-Lundqvist C, et al. Cardiac tachyarrhythmias and patient values and preferences for their management: the European Heart Rhythm Association (EHRA) consensus document endorsed by the Heart Rhythm Society (HRS), Asia Pacific Heart Rhythm Society (APHRS), and Sociedad Latinoamericana de Estimulacion Cardiaca y Electrofisiologia (SOLEACE). *Europace : European pacing, arrhythmias, and cardiac electrophysiology : journal of the working groups on cardiac pacing, arrhythmias, and cardiac cellular electrophysiology of the European Society of Cardiology* 2015;17(12):1747-69. doi: 10.1093/europace/euv233 [published Online First: 2015/06/26]

22. Frankel DS, Parker SE, Rosenfeld LE, et al. HRS/NSA 2014 Survey of Atrial Fibrillation and Stroke: Gaps in Knowledge and Perspective, Opportunities for Improvement. *J Stroke*

For peer review only

Cerebrovasc Dis 2015;24(8):1691-700. doi: 10.1016/j.jstrokecerebrovasdis.2015.06.026 [published Online First: 2015/08/11]

23. McCabe PJ, Schad S, Hampton A, et al. Knowledge and self-management behaviors of patients with recently detected atrial fibrillation. *Heart Lung* 2008;37(2):79-90. doi: 10.1016/j.hrtlng.2007.02.006 [published Online First: 2008/03/29]

24. Desteghe L, Engelhard L, Raymaekers Z, et al. Knowledge gaps in patients with atrial fibrillation revealed by a new validated knowledge questionnaire. *Int J Cardiol* 2016;223:906-14. doi: 10.1016/j.ijcard.2016.08.303 [published Online First: 2016/09/03]

25. Lane DA, Ponsford J, Shelley A, et al. Patient knowledge and perceptions of atrial fibrillation and anticoagulant therapy: effects of an educational intervention programme. The West Birmingham Atrial Fibrillation Project. *Int J Cardiol* 2006;110(3):354-8. doi: 10.1016/j.ijcard.2005.07.031 [published Online First: 2005/10/29]

26. Clarkesmith DE, Pattison HM, Lip GY, et al. Educational intervention improves anticoagulation control in atrial fibrillation patients: the TREAT randomised trial. *PLoS One* 2013;8(9):e74037. doi: 10.1371/journal.pone.0074037 [published Online First: 2013/09/17]

27. Clarkesmith DE, Pattison HM, Borg Xuereb C, et al. Developing a Complex Educational-Behavioural Intervention: The TREAT Intervention for Patients with Atrial Fibrillation. *Healthcare (Basel)* 2016;4(1) doi: 10.3390/healthcare4010010 [published Online First: 2016/07/16]

28. Smith DE, Xuereb CB, Pattison HM, et al. TRial of an Educational intervention on patients' knowledge of Atrial fibrillation and anticoagulant therapy, INR control, and outcome of Treatment with warfarin (TREAT). *BMC Cardiovasc Disord* 2010;10:21. doi: 10.1186/1471-2261-10-21 [published Online First: 2010/05/22]

29. Pattanaphesaj J, Thavorncharoensap M, Ramos-Goni JM, et al. The EQ-5D-5L Valuation study in Thailand. *Expert Rev Pharmacoecon Outcomes Res* 2018;18(5):551-58. doi: 10.1080/14737167.2018.1494574 [published Online First: 2018/06/30]

30. Sonsa-ardjit N, Sakthong P. Reliability and validity of the Thai version of EQ-5D-5L questionnaire on patients with chronic disease. *Chula Med J* 2015;59(5):489-501.

For peer review only

31. Gale NK, Heath G, Cameron E, et al. Using the framework method for the analysis of qualitative data in multi-disciplinary health research. *BMC Med Res Methodol* 2013;13:117. doi: 10.1186/1471-2288-13-117 [published Online First: 2013/09/21]

For peer review only

Figure legend

Figure 1: Study design and recruitment

Group 1: Usual care using warfarin [control] Group 2: Care pathway designated based on stratification of patients using the SAMe-TT_2R_2 score [warfarin plus usual care].

Group 2 care pathways:

Group 2a: SAMe-TT_2R_2 score 0-2 [warfarin plus usual care] NB. Usual care would include management in the anticoagulation clinic, and patient information about AF and need for warfarin by a healthcare professional using the standard warfarin education checklist.

Group 2b: SAMe-TT_2R_2 score >2 group [warfarin plus intensive TREAT education-behavioural intervention as an adjunct to their regular INR monitoring to improve their TTR on warfarin.

Figure 2: Timing of randomization, study visits and follow-up

For peer review only

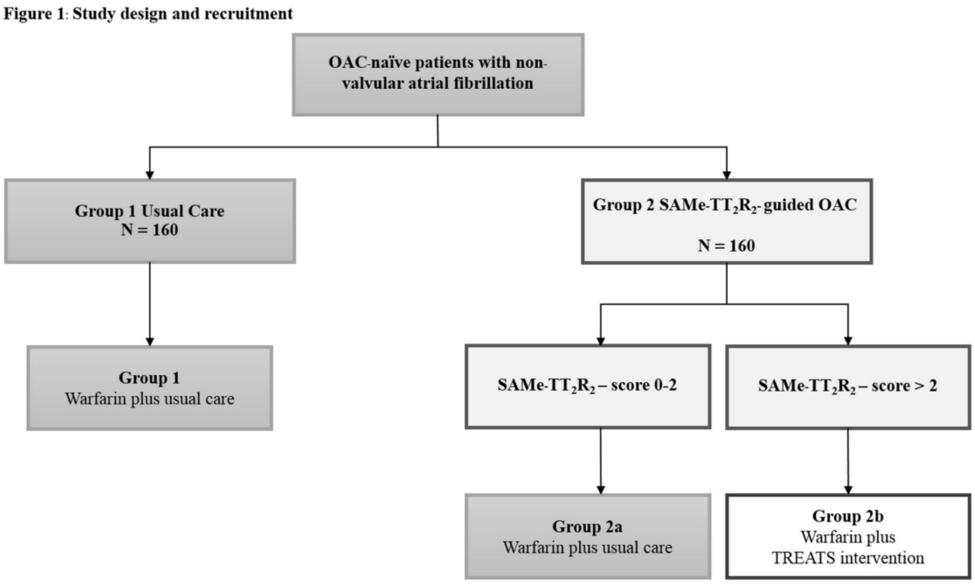

Figure 1: Study design and recruitment
Group 1: Usual care using warfarin [control] Group 2: Care pathway designated based on stratification of patients using the SAMe-TT2R2 score [warfarin plus usual care].
Group 2 care pathways:
Group 2a: SAMe-TT2R2 score 0-2 [warfarin plus usual care] NB. Usual care would include management in the anticoagulation clinic, and patient information about AF and need for warfarin by a healthcare professional using the standard warfarin education checklist.
Group 2b: SAMe-TT2R2 score >2 group [warfarin plus intensive TREAT education-behavioural intervention as an adjunct to their regular INR monitoring to improve their TTR on warfarin.

243x148mm (600 x 600 DPI)

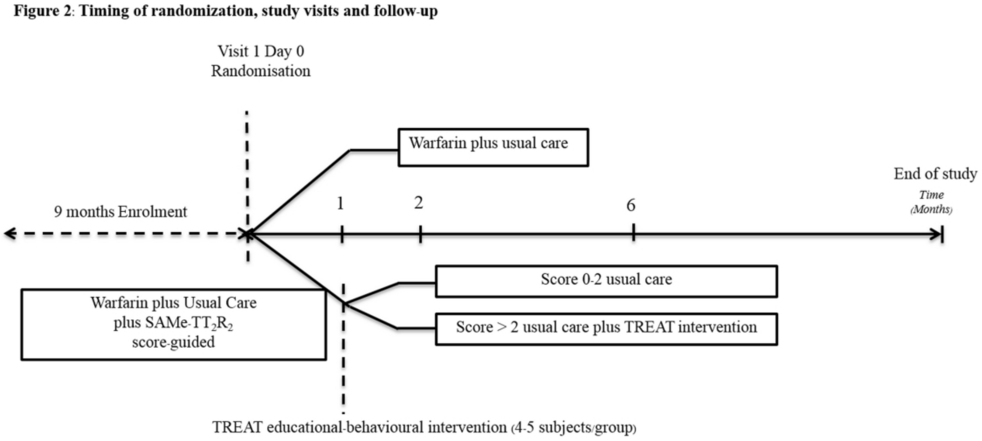

Figure 2: Timing of randomization, study visits and follow-up

129x59mm (600 x 600 DPI)

Appendix (Investigators list)

Trial Management Group of TREATS-AF: Arintaya Phrommintikul, Gregory Lip, G Neil Thomas, Rungroj Krittiyaphong, Wanwarang Wongcharoen, Surakit Nathisuwan, Gemma Slinn, Sukhi Sehmi, Siriluck Gunaparn, Deirdre Lane, Kate Jolly, Susan Jowett, Unchalee Permsuwan, Jonathan Mathers, Wichuda Jiraporncharoen, Chaisiri Angkurawaranon, Anita Slade, Samir Mehta, Antika Wongthanee, Neil Winkle

Trial Steering Committee – TSC: Smonporn Boonyaratavej Songmuang – Chair, Rapeephon Kunjara Na Ayudhya, Lin Yenn-Jiang, Tomorn Thongsri

Data Monitoring and Ethics Committee – DMEC: Tippawan Liabsuetrakul, Kemmawadee Preedalikit, Thoranis Chantrarat,

Participating local investigator list of TREATS-AF;

1. Chiangrai Prachanukroh Hospital: Wattana Wongtheptien, Suparat Wattanasombat, Jutarat Kaewdam, Nongluk Mananusorn, Wuttichai Sawatna

2. Nakornping Hospital: Thanyaluck Chotayaporn, Kultida Lertthanaphol, Busarakam Pothongsunun, Jirarat Chandee, Tananya Prasit, Chotika Ruttanapornnukul, Ketsanee Sripattanatrakul, Vullida Cheingyothakul

3. Maharaj Nakorn Chiang Mai Hospital: Narawudt Prasertwitayakij, Kanokporn Niwatananun, Voratima Silavanich, Mantiwee Nimworapan, Phornwinee Somsap, Tikumporn Pornwisrtsirikul, Praphaphan Daoram, Chayanit Srisongmuang

4. Lampang Hospital: Natrawee Bureekam, Siriporn Intharangsri, Chonlada Kawila

5. Siriraj Hospital: Arjbordin Winigkul, Yodying Kaolawanich, Ajjma Sarapakdi, Usanee Pienpattapong, Olaree Chaiphet

For peer review only

6. Maharat Nakhon Ratchasima Hospital: Bancha Sookananchai, Piti Niyomsiriwanich, Weerapan Wiwatworapan, Ubonwan Sapoo, Suttiwanik Kowitphattana, Ketmanee Sae-Ueng, Arisorn Jirapermpun

7. Srinagarind Hospital and Queen Sirikit Heart Center: Vichai Senthong, Supaporn Onsanit, Siriporn Jantharuechai, Wachiraya Tipboonchu

For peer review only

STANDARD PROTOCOL ITEMS: RECOMMENDATIONS FOR INTERVENTIONAL TRIALS

SPIRIT 2013 Checklist: Recommended items to address in a clinical trial protocol and related documents*

Section/item	Item No	Description	Addressed on page number
**Administrative information**			
Title	1	Descriptive title identifying the study design, population, interventions, and, if applicable, trial acronym	1
Trial registration	2a	Trial identifier and registry name. If not yet registered, name of intended registry	2
	2b	All items from the World Health Organization Trial Registration Data Set	2
Protocol version	3	Date and version identifier	8
Funding	4	Sources and types of financial, material, and other support	19
Roles and responsibilities	5a	Names, affiliations, and roles of protocol contributors	19
	5b	Name and contact information for the trial sponsor	19
	5c	Role of study sponsor and funders, if any, in study design; collection, management, analysis, and interpretation of data; writing of the report; and the decision to submit the report for publication, including whether they will have ultimate authority over any of these activities	19

For peer review only

	5d	Composition, roles, and responsibilities of the coordinating centre, steering committee, endpoint adjudication committee, data management team, and other individuals or groups overseeing the trial, if applicable (see Item 21a for data monitoring committee)	Appendix
**Introduction**			
Background and rationale	6a	Description of research question and justification for undertaking the trial, including summary of relevant studies (published and unpublished) examining benefits and harms for each intervention	5-6
	6b	Explanation for choice of comparators	5-6
Objectives	7	Specific objectives or hypotheses	6
Trial design	8	Description of trial design including type of trial (eg, parallel group, crossover, factorial, single group), allocation ratio, and framework (eg, superiority, equivalence, noninferiority, exploratory)	6
**Methods: Participants, interventions, and outcomes**			
Study setting	9	Description of study settings (eg, community clinic, academic hospital) and list of countries where data will be collected. Reference to where list of study sites can be obtained	7-8
Eligibility criteria	10	Inclusion and exclusion criteria for participants. If applicable, eligibility criteria for study centres and individuals who will perform the interventions (eg, surgeons, psychotherapists)	8-9
Interventions	11a	Interventions for each group with sufficient detail to allow replication, including how and when they will be administered	9-10
	11b	Criteria for discontinuing or modifying allocated interventions for a given trial participant (eg, drug dose change in response to harms, participant request, or improving/worsening disease)	15

	11c	Strategies to improve adherence to intervention protocols, and any procedures for monitoring adherence (eg, drug tablet return, laboratory tests)	9
	11d	Relevant concomitant care and interventions that are permitted or prohibited during the trial	8
Outcomes	12	Primary, secondary, and other outcomes, including the specific measurement variable (eg, systolic blood pressure), analysis metric (eg, change from baseline, final value, time to event), method of aggregation (eg, median, proportion), and time point for each outcome. Explanation of the clinical relevance of chosen efficacy and harm outcomes is strongly recommended	9
Participant timeline	13	Time schedule of enrolment, interventions (including any run-ins and washouts), assessments, and visits for participants. A schematic diagram is highly recommended (see Figure)	11
Sample size	14	Estimated number of participants needed to achieve study objectives and how it was determined, including clinical and statistical assumptions supporting any sample size calculations	13
Recruitment	15	Strategies for achieving adequate participant enrolment to reach target sample size	13

Methods: Assignment of interventions (for controlled trials)

Allocation:

Sequence generation	16a	Method of generating the allocation sequence (eg, computer-generated random numbers), and list of any factors for stratification. To reduce predictability of a random sequence, details of any planned restriction (eg, blocking) should be provided in a separate document that is unavailable to those who enrol participants or assign interventions	8
Allocation concealment mechanism	16b	Mechanism of implementing the allocation sequence (eg, central telephone; sequentially numbered, opaque, sealed envelopes), describing any steps to conceal the sequence until interventions are assigned	11
Implementation	16c	Who will generate the allocation sequence, who will enrol participants, and who will assign participants to interventions	8

Blinding (masking)	17a	Who will be blinded after assignment to interventions (eg, trial participants, care providers, outcome assessors, data analysts), and how	8
	17b	If blinded, circumstances under which unblinding is permissible, and procedure for revealing a participant's allocated intervention during the trial	8

Methods: Data collection, management, and analysis

Data collection methods	18a	Plans for assessment and collection of outcome, baseline, and other trial data, including any related processes to promote data quality (eg, duplicate measurements, training of assessors) and a description of study instruments (eg, questionnaires, laboratory tests) along with their reliability and validity, if known. Reference to where data collection forms can be found, if not in the protocol	11
	18b	Plans to promote participant retention and complete follow-up, including list of any outcome data to be collected for participants who discontinue or deviate from intervention protocols	13
Data management	19	Plans for data entry, coding, security, and storage, including any related processes to promote data quality (eg, double data entry; range checks for data values). Reference to where details of data management procedures can be found, if not in the protocol	13
Statistical methods	20a	Statistical methods for analysing primary and secondary outcomes. Reference to where other details of the statistical analysis plan can be found, if not in the protocol	17
	20b	Methods for any additional analyses (eg, subgroup and adjusted analyses)	16-17
	20c	Definition of analysis population relating to protocol non-adherence (eg, as randomised analysis), and any statistical methods to handle missing data (eg, multiple imputation)	17-18

Methods: Monitoring

Data monitoring	21a	Composition of data monitoring committee (DMC); summary of its role and reporting structure; statement of whether it is independent from the sponsor and competing interests; and reference to where further details about its charter can be found, if not in the protocol. Alternatively, an explanation of why a DMC is not needed	13
	21b	Description of any interim analyses and stopping guidelines, including who will have access to these interim results and make the final decision to terminate the trial	14
Harms	22	Plans for collecting, assessing, reporting, and managing solicited and spontaneously reported adverse events and other unintended effects of trial interventions or trial conduct	13
Auditing	23	Frequency and procedures for auditing trial conduct, if any, and whether the process will be independent from investigators and the sponsor	13
**Ethics and dissemination**			
Research ethics approval	24	Plans for seeking research ethics committee/institutional review board (REC/IRB) approval	8
Protocol amendments	25	Plans for communicating important protocol modifications (eg, changes to eligibility criteria, outcomes, analyses) to relevant parties (eg, investigators, REC/IRBs, trial participants, trial registries, journals, regulators)	18
Consent or assent	26a	Who will obtain informed consent or assent from potential trial participants or authorised surrogates, and how (see Item 32)	9
	26b	Additional consent provisions for collection and use of participant data and biological specimens in ancillary studies, if applicable	-
Confidentiality	27	How personal information about potential and enrolled participants will be collected, shared, and maintained in order to protect confidentiality before, during, and after the trial	13

er review only

Declaration of interests	28	Financial and other competing interests for principal investigators for the overall trial and each study site	19
Access to data	29	Statement of who will have access to the final trial dataset, and disclosure of contractual agreements that limit such access for investigators	19
Ancillary and post-trial care	30	Provisions, if any, for ancillary and post-trial care, and for compensation to those who suffer harm from trial participation	-
Dissemination policy	31a	Plans for investigators and sponsor to communicate trial results to participants, healthcare professionals, the public, and other relevant groups (eg, via publication, reporting in results databases, or other data sharing arrangements), including any publication restrictions	18
	31b	Authorship eligibility guidelines and any intended use of professional writers	18
	31c	Plans, if any, for granting public access to the full protocol, participant-level dataset, and statistical code	18
**Appendices**			
Informed consent materials	32	Model consent form and other related documentation given to participants and authorised surrogates	Inform Consent
Biological specimens	33	Plans for collection, laboratory evaluation, and storage of biological specimens for genetic or molecular analysis in the current trial and for future use in ancillary studies, if applicable	-

*It is strongly recommended that this checklist be read in conjunction with the SPIRIT 2013 Explanation & Elaboration for important clarification on the items. Amendments to the protocol should be tracked and dated. The SPIRIT checklist is copyrighted by the SPIRIT Group under the Creative Commons "Attribution-NonCommercial-NoDerivs 3.0 Unported" license.

For peer review only - http://bmjopen.bmj.com/site/about/guidelines.xhtml

	STUDY PERIOD					
	Enrolment and Allocation	Post-allocation				
**TIMEPOINT****	***t_0*** ***Baseline***	***t_1*** ***1 month***	***t_2*** ***2 month***	***t_3*** ***3 month***	***t_4*** ***6 month***	***t_5*** ***12 month***
**ENROLMENT:**						
**Eligibility screen**	X					
**Informed consent**	X					
**Allocation**	X					
**INTERVENTIONS:**						
Standard of Care		X				
TREAT intervention		X				
**ASSESSMENTS:**						
CLINICAL ASSESSMENT		X	X	X	X	X
ECG		X				
INR		X	X	X	X	X
STANDARD OAC THERAPY EDUCATION		X				
TREAT intervention			X			
QUESTIONNAIRES		X			X	X
QUALITATIVE ASSESSMENT		X			X	
HEALTH ECONOMIC ASSESSMENT		X			X	X
FOLLOW-UP OF CLINICAL EVENTS					X	X

116x160mm (300 x 300 DPI)
